# Feasibility analysis of China's medical insurance coverage of assisted reproductive technology

**DOI:** 10.1038/s41598-024-58640-4

**Published:** 2024-04-05

**Authors:** Rong Huang, Jing-Yun Yu, Wei-Chao He, Ri-Hui Liu

**Affiliations:** 1 Department of Laboratory, Panyu Hexian Memorial Hospital of Guangzhou, Guangzhou, 511400 China; 2 Department of Health Care, Dongguan Maternal and Child Health Care Hospital, Dongguan, 523112 China; 3 Medical Insurance Office, Human Resources and Social Security Bureau of Guangzhou’s Nansha District, No.15 Huanshi Avenue Middle, Nansha District, Guangzhou City, 511466 Guangdong China

**Keywords:** Medical insurance, Assisted reproductive technology, Infertility, Cost, Population, Health care economics, Health policy, Health services, Public health

## Abstract

There are millions of patients experiencing infertility in China, but assisted reproductive technology (ART) is performed at the patient's expense and is difficult to afford. With the sharp decline in China's birth rate, there is a growing controversy over the inclusion of ART in medical insurance (MI). This study aims to explore the feasibility of ART coverage by MI for the first time. We obtained basic data such as the prevalence of infertility, the cost of ART, and the success rate in China with the method of meta-analysis and consulting the government bulletin. Then, we calculated the number of infertile couples in China and the total financial expenditure of MI covering ART. Finally, we discussed the feasibility of coverage, and analyzed the population growth and economic benefits after coverage. According to our research results, it was estimated that there were 4.102–11.792 million infertile couples in China, with an annual increase of 1.189–1.867 million. If MI covered ART, the fund would pay 72.313–207.878 billion yuan, accounting for 2–6% of the current fund balance, and the subsequent annual payment would be 20.961–32.913 billion yuan, accounting for 4–7% of the annual fund balance. This was assuming that all infertile couples would undergo ART, and the actual cost would be lower. The financial input‒output ratio would be 13.022. Benefiting from the inclusion of ART in MI coverage, there would be 3.348–9.624 million new live infants, and 8–13% newborns would be born every year thereafter, which means that by 2050, 37–65 million people would be born. Due to its affordable cost, high cost-effectiveness and favourable population growth, it may be feasible to include ART in MI.

## Introduction

In recent years, China seems to have suffered from a population crisis, and the birth rate has dropped sharply. At the same time, the trend of late childbearing is becoming increasingly obvious. The average age of first childbearing for Chinese women increased from 24.3 years in 2006 to 26.9 years in 2016^1^, and the delivery rate of pregnant women over 35 years old in tertiary hospitals increased from 12.54% in 2015 to 17.43% in 2017^2^. Advanced female age is an important risk factor for infertility^3^, and the continuous decline in male reproductive health exacerbates this risk^4^. According to the World Health Organization (WHO), infertility is a reproductive system disease defined as the inability to achieve clinical pregnancy after 12 months or more of regular unprotected sexual intercourse^5^. According to research reports, the proportion of infertile couples in China has increased from 6.89 to 12.5% over the last 20 years^6^, and China's fertility rate is declining and is expected to decline from its peak of 1.75 in 2016 to around 1.3 in 2050^7^. As an important treatment for infertility, assisted reproductive technology (ART) has developed rapidly in China. In 2016, the number of ART cycles exceeded 1 million^8^. However, due to the high cost and lack of inclusion in medical insurance (MI), more than 60% of infertile couples cannot afford it^2,9,10^.

Currently, over 80% of European and Oceanian countries provide partial or full reimbursement for fertility treatment^11^. In China, whether ART needs to be included in MI is highly controversial. For the sake of cost, the Chinese government has always held a negative attitude towards this. With the implementation of China's three-child policy in 2021, the dispute has become increasingly serious. So far, to our knowledge, only Lin Wang^12^ has published a review on the feasibility of MI incorporating ART. However, this article did not calculate the size and total treatment cost of the infertile population. The existing surveys lack a unified definition of infertility, resulting in great differences in prevalence^5^. The composition of ART costs reported in different documents is also different. These make it difficult to calculate the total cost of incorporating ART into MI. Therefore, in this article, we discussed for the first time the total financial cost and feasibility of incorporating ART into MI and analysed the population increment and economic benefits after its inclusion. We hope to provide some positive clues to encourage the government to incorporate ART into MI, and also hope to provide some references for policy makers in other countries with the same situation as China.

## Material and methods

### Research design

We obtained basic data through meta-analysis and government bulletins. Then we use the mathematical model to calculate the total cost of incorporating ART into MI and the resulting population growth. Specifically, it is divided into the following three steps:

Part 1: We collected literature and conducted a meta-analysis to obtain data on the prevalence of infertility, the success rate of ART, and the cost of ART in China. As a supplement, the data of "the Chinese Society of Reproductive Medicine (CSRM) data reporting system" built by the Chinese Reproductive Medicine Association were also collected^13–15^. We drew a decision tree diagram of the success rate and cost per complete cycle. We also collected the cost per live birth and MI coverage in other countries.

Part 2: According to the infertility rate results in the first step, we used the total number of women aged 20–49 years in the seventh census data (The Chinese government conducts a comprehensive census of the country's population every ten years and the seventh census was in 2020) to predict the number of infertile couples and used the female age-specific fertility rate to predict the number of new infertile couples each year. We drew a statistical map of the economic burden of ART and MI coverage in various countries for comparison with China.

Part 3: We calculated the total cost of ART for all infertile couples. According to the payment proportion and fund balance of MI, we estimated the financial expenditure required to include ART in MI and evaluated the feasibility. We conducted a cost‒benefit analysis incorporating ART into MI and predicted the population from 2022 to 2050 with or without MI coverage.

### Important definitions

1. ART: ART is divided into invitro fertilization (IVF), intracytoplasmic sperm injection (ICSI), preimplantation genetic diagnosis (PGD/PGS) and artificial insemination (AI). Compared with the other types, AI has the lowest cost and lowest proportion^14,15^. Therefore, in this paper, ART did not include AI.
2. Infertility: According to the WHO, infertility is defined as the failure to achieve clinical pregnancy after 12 months or more of regular unprotected sexual intercourse^16^. However, some guidelines define the duration of unprotected sex as 2 years^5^. In this paper, we used "one-year infertility" and "two-year infertility" to represent these two definitions, respectively.
3. Cycle: Each fresh embryo or frozen embryo transfer is recorded as a "transfer cycle". A "complete cycle" refers to the completion of fresh embryo transfer and all subsequent frozen embryo transfers after one ovarian stimulation cycle; an "initiated cycle" only involves the first transfer.
4. Live birth: The delivery of twins and above is only recorded as one live birth, and only the first live birth cycle is recorded for each person. The live birth rate (LBR) is calculated as follows: LBR = (Number of live births)/(Total number of people receiving treatment); Cumulative live birth rate (CLBR) = (Total number of live births after one or more treatments)/(The total number of people who have received treatment in the same period). "The optimistic estimate of the CLBR" means that patients who still have frozen embryos that were not transferred or have not returned to the hospital for a follow-up treatment cycle have the same opportunity to obtain live births as patients who continue treatment.
5. ART costs: ART costs refer to the expenses of both spouses, include direct medical costs (infertility evaluations, imaging and laboratory examinations, medication treatments, and ART treatments including sperm or ovum procurement), other medical expenses (physical examination costs before the cycle, complication treatment, pregnancy and childbirth costs), and indirect costs (food, accommodation, lost wages and transportation costs). The costs in our article only include direct medical costs for both spouses.
6. Couples: "All couples" refer to all couples whose wives are between 20 and 49 years old. "Couples at risk" refer to newlyweds or couples planning a pregnancy.

### Literature search

We conducted a literature search on four topics in Chinese and English databases: the "Prevalence of infertility in China" (Topic A), "Success rate of ART in China" (Topic B), "Cost of ART per live birth in China" (Topic C), and "Cost of ART per live birth in various countries" (Topic D). The search period was from January 2000 to January 2022. Limited by the length of the article, we submitted detailed information on the literature search in Supplementary material 1, including the search strategies, search terms, quality evaluation, inclusion and exclusion criteria, and extraction contents. There were also many important indicators, such as the birth rate, annual MI expenditure, and gross domestic product (GDP), which came from the data published on the government's official website.

### Statistical analyses

We used Stata software (version 12.0) to conduct meta-analysis on Topic A, Topic B and Topic C. The I2 statistic test was used to test heterogeneity. When I2 was > 50%, a random effect model was adopted; otherwise, a fixed effect model was adopted. Begg's test was used to evaluate publication bias. For Topic D, meta-analysis was not possible because some studies did not provide their sample size and standard deviation, and we used the average instead. Since the direct medical cost has not increased significantly in the past 20 years, we calculated the disease economic burden by dividing the cost per live birth by the per capita GDP in 2020. Based on population data from China from 1981 to 2021, we used neural network models to predict the population trends from 2022 to 2050 with and without ART being included in MI, and then estimated the promoting effect of the inclusion on the population. The decision tree model was drawn by TreeAge (2011 version) software. ArcGIS software (version 10.6) was used to draw a statistical map. The neural network model was analyzed using MATLAB (2014 version) software. The lifelong economic value (LEV)^17^, net tax contribution^18^, and financial input‒output ratio^18^ were used as evaluation indicators of economic benefits.

### Ethics and consent to participate

This study did not require ethical review.

## Results

### Part 1

For Topic A, among all couples, the 1-year infertility rate was 0.042 (0.038–0.046), and the two-year infertility rate was 0.015 (0.013–0.018). Among couples at risk, the one-year infertility rate was 0.153 (0.133–0.172) and the two-year infertility rate was 0.089 (0.068–0.110). The composition of infertility factors is 60% for women, 20% for men, and 20% for both parties or unclear factors. In addition, the visit rate of infertile couples was 41.4% (27.5–55.3%), of which the treatment rate of hospitals above the municipal level that may have ART qualifications was 24.8% (7.6–42.0%).

**Figure 1:**
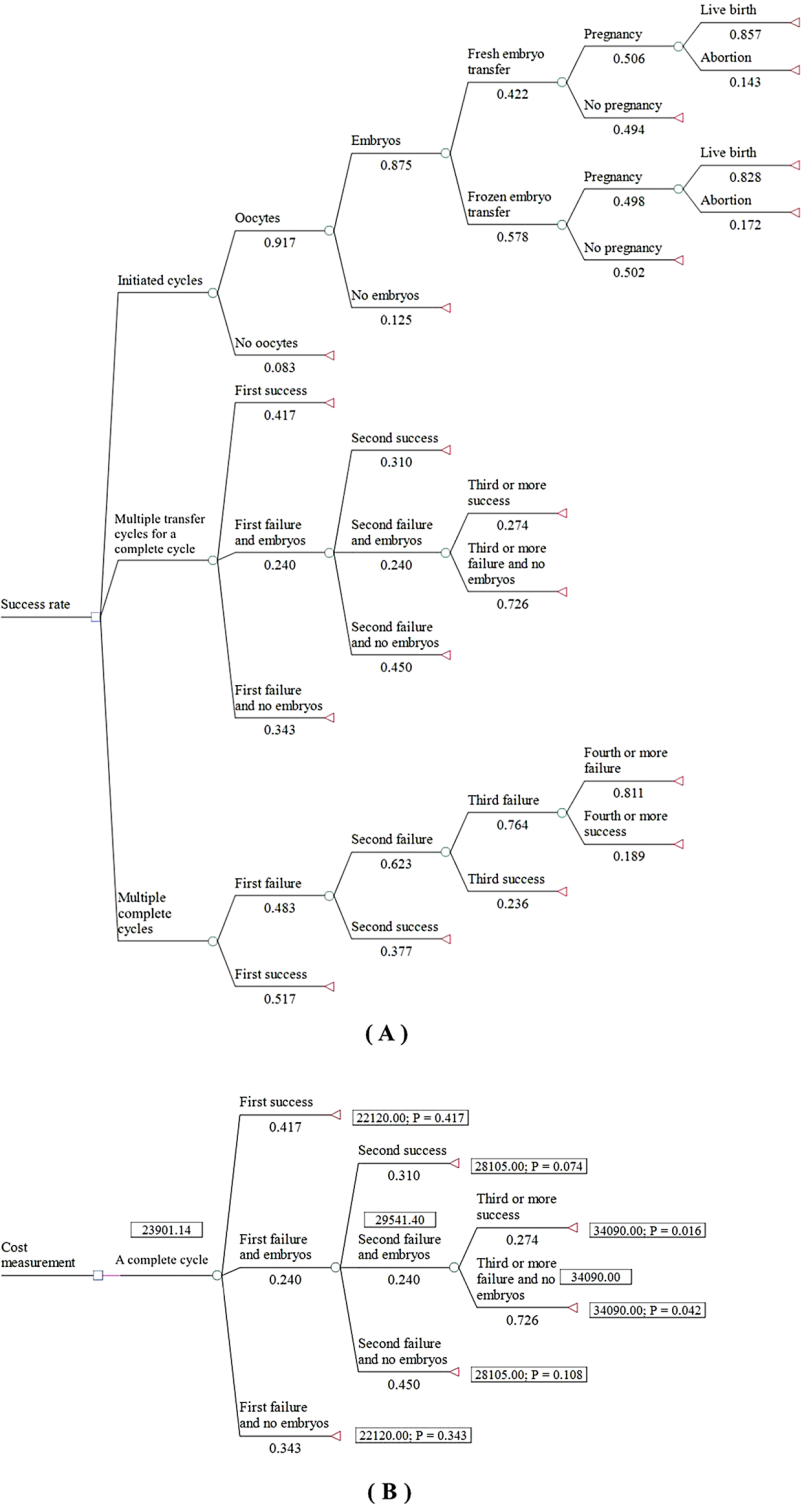
Decision tree of the success rate and complete cycle cost (**A**—Success rate; **B**—Complete cycle cost).

For Topic B, a meta-analysis was used, and the data from the CSRM system from 2013 to 2018 were collected^13–15^. Based on these results, we drew a decision tree diagram of the ART success rate (Fig. 1A) and calculated the LBR of three cases in China. In an initiated cycle, there was a 14.7% possibility of obtaining live birth by transplanting fresh embryos and a 19.1% possibility of obtaining live birth by transplanting frozen embryos. In a complete cycle, there was a cumulative 50.7% possibility of obtaining live birth. After four complete cycles, the optimistic estimate of the CLBR was 81.4%.

For Topic C, through meta-analysis, we found that the cost per live birth was 67, 002 yuan, and the cost per complete cycle was estimated to be 23, 901 yuan by the decision tree model (Fig. 1B). ART treatments account for approximately 50% of the total cost, medical treatments account for 40%, and B-ultrasonic examination accounts for 10%. The cost list can be found in Supplementary Material 4.

For Topic D, a total of 38 articles covering 24 countries were collected.

The results of Topics A, B and C are shown in Table 1. Limited by the number of words, the study selection flow chart is shown in Supplementary material 2, and the literature information is shown in Supplementary material 3. In addition, we retrieved two reviews, including the status of ART covered by MI in various countries^11,19^.

**Table 1:** Results of the meta-analysis and CSRM system collection.

Group	Number of studies	Sample size	Analyzed value
Topic A: Prevalence of infertility in China			Infertility rate (95%CI)
Entire couples (female age 20–49) of one-year infertility	19	8,022,162	0.042 (0.038–0.046)
Entire couples (female age 20–49) of two-year infertility	6	6,023,427	0.015 (0.013–0.018)
Risk couples (female age 20–49) of one-year infertility	25	266,662	0.153 (0.133–0.172)
Risk couples (female age 20–49) of two-year infertility	9	160,256	0.089 (0.068–0.110)
Entire couples (female age 20–24) of one-year infertility	6	20,383	0.045 (0.021–0.069)
Entire couples (female age 25–29) of one-year infertility	6	8351	0.047 (0.029–0.064)
Entire couples (female age 30–34) of one-year infertility	5	5607	0.048 (0.025–0.070)
Entire couples (female age 35–39) of one-year infertility	4	5184	0.035 (0.018–0.052)
Entire couples (female age 40–49) of one-year infertility	5	5069	0.032 (0.010–0.054)
Entire couples (female age 20–24) of two-year infertility	3	22,644	0.025 (0.023–0.027)*
Entire couples (female age 25–29) of two-year infertility	3	81,304	0.018 (0.013–0.024)
Entire couples (female age 30–34) of two-year infertility	3	116,933	0.011 (0.010–0.012)*
Entire couples (female age 35–39) of two-year infertility	3	369,540	0.011 (0.006–0.017)
Entire couples (female age 40–49) of two-year infertility	3	371,238	0.010 (0.006–0.015)
Risk couples (female age 20–24) of one-year infertility	11	22,538	0.143 (0.111–0.174)
Risk couples (female age 25–29) of one-year infertility	8	25,740	0.148 (0.108–0.189)
Risk couples (female age 30–34) of one-year infertility	10	16,396	0.165 (0.115–0.215)
Risk couples (female age 35–39) of one-year infertility	9	26,180	0.209 (0.159–0.259)
Risk couples (female age 40–49) of one-year infertility	10	22,256	0.220 (0.161–0.280)
Risk couples (female age 20–24) of two-year infertility	3	45,152	0.090 (0.047–0.134)
Risk couples (female age 25–29) of two-year infertility	3	23,189	0.103 (0.066–0.140)
Risk couples (female age 30–34) of two-year infertility	2	13,466	0.123 (0.097–0.148)
Risk couples (female age 35–39) of two-year infertility	2	13,541	0.132 (0.126–0.137)*
Risk couples (female age 40–49) of two-year infertility	2	7366	0.126 (0.098–0.153)
Topic B: Success rate of ART in China			Live birth rate (95%CI)
Livebirth rate of Meta			
First complete cycle	4	75,177	0.517 (0.431–0.604)
Second complete cycle	4	14,466	0.377 (0.333–0.421)
Third complete cycle	4	3530	0.236 (0.148–0.324)
Fourth complete cycle	4	1412	0.189 (0.169–0.210)*
First transfer cycle of a complete cycle	3	34,918	0.417 (0.395–0.438)
Second transfer cycle of a complete cycle	3	5053	0.310 (0.165–0.454)
≥ Third transfer cycles of a complete cycle	3	1176	0.274 (0.121–0.428)
Livebirth rate of CSRM			
Transfer cycle	–	1,583,021	0.404 (–)
Fresh embryo transfer	–	667,542	0.419 (–)
Frozen embryo transfer	–	915,479	0.393 (–)
Transfer cycle of 18–34 women	–	622,066	0.455 (–)
Transfer cycle of 35–40 women	–	197,196	0.318 (–)
Transfer cycle of 41–44 women	–	39,531	0.119 (–)
Transfer cycle of 45–49 women	–	6916	0.042 (–)
Multiple birth rate	–	638,802	0.254 (–)
Topic C: Cost per live birth of ART in China^^^			Cost (95%CI)
Per live birth	21	21,567	67,002 (62,101–71,903)
Per initiated cycle	21	21,567	22,120 (20,512–23,729)
Per frozen-thawed embryo transfer cycle	2	1551	5985 (2383–9586)
Per live birth of GnRH-a long protocol	16	15,494	61,866 (56,273–67,459)
Per live birth of GnRH-ant protocol	11	4408	63,468 (50,504–76,433)
Per live birth of minimal-stimulation protocol	6	593	70,006 (51,732–88,280)
Per live birth of PPOS protocol	3	292	70,072 (3905–136,239)
Per live birth of natural cycle protocol	1	374	269,912 (–)
Per live birth of 20–34 years old	7	12,189	54,165 (46,728–61,601)^#^
Per live birth of 35–49 years old	8	4646	89,935 (75,239–104,632)
Per live birth of 2000–2010	3	360	63,934 (42,348–85,519)^#^
Per live birth of 2011–2021	18	21,207	67,376 (62,230–72,521)
Per initiated cycle of GnRH-a long protocol	16	15,494	25,140 (22,967–27,313)
Per initiated cycle of GnRH-ant protocol	11	4408	21,883 (19,464–24,301)
Per initiated cycle of minimal-stimulation protocol	6	593	16,178 (12,167–20,190)
Per initiated cycle of PPOS protocol	3	292	14,496 (7443–21,550)
Per initiated cycle of natural cycle protocol	1	374	12,254 (–)
Per initiated cycle of 20–34 years old	7	12,189	22,662 (20,651–24,673)
Per initiated cycle of 35–49 years old	8	4646	21,004 (19,158–22,851)
Per initiated cycle of 2000–2010	3	360	19,155 (10,486–27,824)
Per initiated cycle of 2011–2021	18	21,207	22,469 (20,790–24,149)

(1) GnRH—Gonadotropin releasing hormone; PPOS—Progestin primed ovarian stimulation. (2) *Fixed effect model, ^#^ Begg's test shows that there is publication bias. (3) ^^^Including the costs of both spouses during the ART procedure.

### Part 2

**Figure 2:**
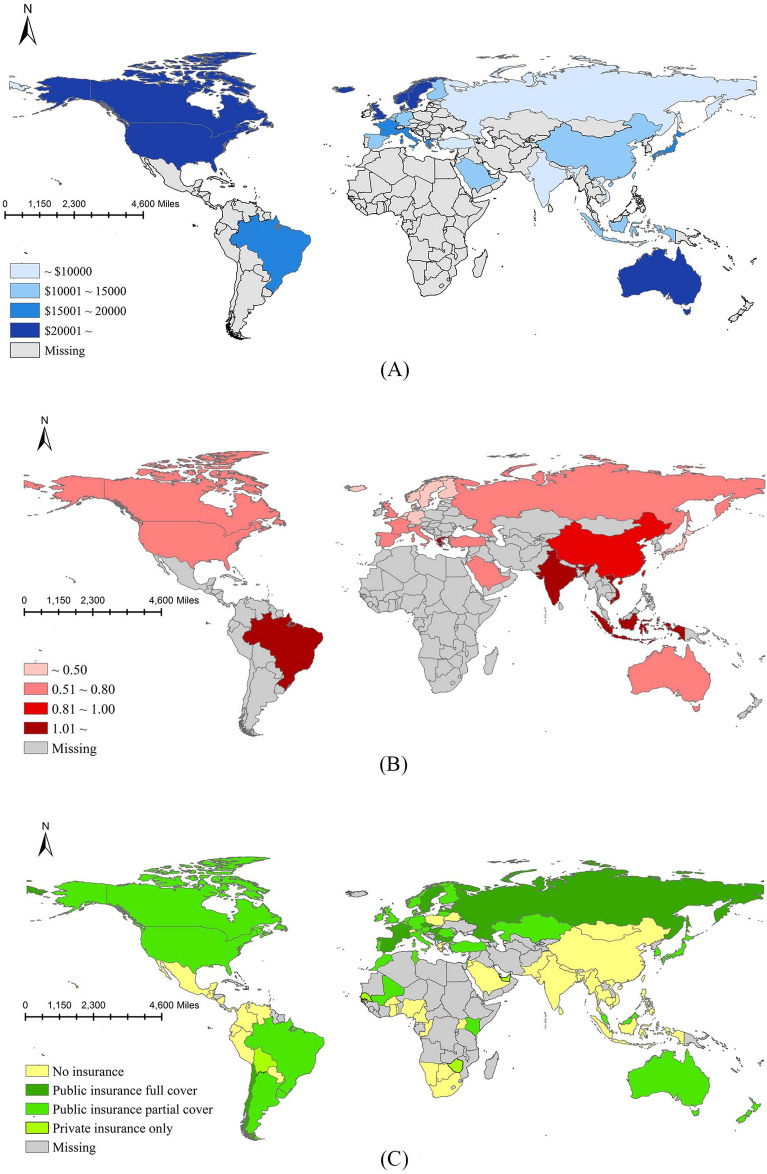
ART cost per live birth and the inclusion of ART costs in MI (**A**—The ART cost per live birth; **B**—Ratio of the ART cost per live birth to the GDP per capita; **C**—The inclusion of ART costs in MI; The statistical map was created by the author using ArcGIS software (version 10.6), https://www.esri.com/en-us/arcgis/products/index).

The results are shown in Table 2. The calculated number of infertile patients remained stable before and after age adjustment. It was estimated that there were 4.102 (2-year infertility) ~ 11.792 million (1-year infertility) infertile couples in China, with an annual increase of 1.189 (2-year infertility) ~ 1.867 million (1-year infertility). The average ART cost per live birth of each country is shown in Fig. 2A, the economic burden is shown in Fig. 2B, and the MI coverage is shown in Fig. 2C.

**Table 2:** Estimation of the number of infertile couples in China.

Group	Total number (1000)	1-year infertility rate	1-year infertility number (1000)	2-year infertility rate	2-year infertility number (1000)
^a^Entire couples (female age 20–49)					
No adjustment	288,661	0.042	12,124	0.015	4330
Adjusted for female age					
20–24	35,266	0.045	1,587	0.025	882
25–29	43,685	0.047	2,053	0.018	786
30–34	60,273	0.048	2,893	0.011	663
35–39	48,081	0.035	1,683	0.011	529
40–49	101,356	0.032	3,243	0.010	1014
Total	288,661	–	11,459	–	3873
^c^Average	–	–	11,792	–	4102
^b^Risk couples (female age 20–49)					
No adjustment	11,864	0.153	1,815	0.089	1056
Adjusted for female age					
20–24	1,947	0.143	278	0.090	175
25–29	4,324	0.148	640	0.103	445
30–34	3,921	0.165	647	0.123	482
35–39	1,294	0.209	270	0.132	171
40–49	378	0.220	83	0.126	48
Total	11,864	–	1,919	–	1321
^c^Average	–	–	1,867	–	1189

^a^According to the female age group population data in the seventh population census.

^b^Inferring from the age-specific fertility rate of women in the data of the seventh population census.

^c^Taking the average value before and after adjustment as the final estimated value.

### Part 3

We calculated the total cost of ART for all infertile couples using 23, 901 yuan per complete cycle and 67, 002 yuan per live birth and then took the average of the two as the final estimate. We found that MI needed to spend 210.519 (2-year infertility) ~ 605.178 billion yuan (1-year infertility) and then spend 61.021 (2-year infertility) ~ 95.816 billion yuan (one-year infertility) every year. Since the establishment of the National Healthcare Security Administration (NHSA) in 2018, the price of new drugs through MI has decreased by more than 50% on average, and the average payment proportion of MI funds is 68.7%. According to this standard, if ART is included in MI, the fund would pay 72.313 (2-year infertility) ~ 207.878 (1-year infertility) billion yuan, and the subsequent annual payment would be 20.961 (2-year infertility) ~ 32.913 (1-year infertility) billion yuan. The cost paid by patients themselves would be reduced to nearly one-sixth of the original cost. These results are shown in Table 3.

**Table 3:** Financial expenses and cost-effectiveness after the inclusion of ART in MI.

Index		Current number of infertile couples		New infertile couples per year	
		11.792 million	4.102 million	1.867 million	1.189 million
IVF	Complete cycles (million)	23.747	8.261	3.760	2.394
	Live births (million)	9.593	3.337	1.519	0.967
	Live infants (million)	12.030	4.185	1.905	1.213
Current cost	According to live birth (billion ¥)	642.780	223.599	101.770	64.812
	According to complete cycle (billion ¥)	567.575	197.438	89.863	57.229
	Average of the above two (billion ¥)	605.178	210.519	95.816	61.021
Expenses after inclusion in MI	Cost after MI negotiation (billion ¥)	302.589	105.259	47.908	30.510
	MI payment (billion ¥)	207.878	72.313	32.913	20.961
	Personal payment (billion ¥)	94.710	32.946	14.995	9.550
Cost-effectiveness	New live infants due to MI (million)	9.624	3.348	1.524	0.970
	LEV per complete cycle (million ¥)	3.174	3.174	3.174	3.174
	Net tax contribution (billion ¥)	2707.094	941.698	428.608	272.959
	Financial input–output ratio	13.022	13.022	13.022	13.022

(1) ART—Assisted reproductive technology, MI—medical insurance, LEV—lifetime economic value. (2) Assuming that all infertile couples receive ART, and the losers enter the next initiated cycle (up to 4 times); In this case, the cumulative live birth rate is 81.36%, the average number of complete cycle is 2.014, and LBR per complete cycle is 0.404. (3) Live infants = Live births * (1 + Multiple birth rate 0.254). (4) LEV per complete cycle = Per capita GDP 80, 976 ¥ * Life expectancy 77.3 years * Live infants per complete cycle (0.404 * 1.254); Net tax contribution = Total tax amount (Per capita tax 12, 235 ¥ * Life expectancy 77.3 years)—Educational financial investment (Per capita education funds 15, 280 ¥ * Per capita education years 10.9 years)—Medical financial investment (Per capita medical cost 1, 760 ¥ * Life expectancy 77.3 years)—Pension financial investment (Per capita pension cost 20, 496 ¥ * Years of pension 17.3 years)—MI payment on ART; Financial input–output ratio = Net tax contribution / MI payment; The data are the values in 2021 and are derived from the official government website. (5) Assuming that after ART is included, the total cost will be reduced by 50% and the fund will pay 68.7%. (6) Assuming that at present 20% of infertility couples receive ART. (7) The cost-effectiveness analysis does not consider the impact of changes in per capita GDP, life expectancy, years of education and inflation.

**Figure 3:**
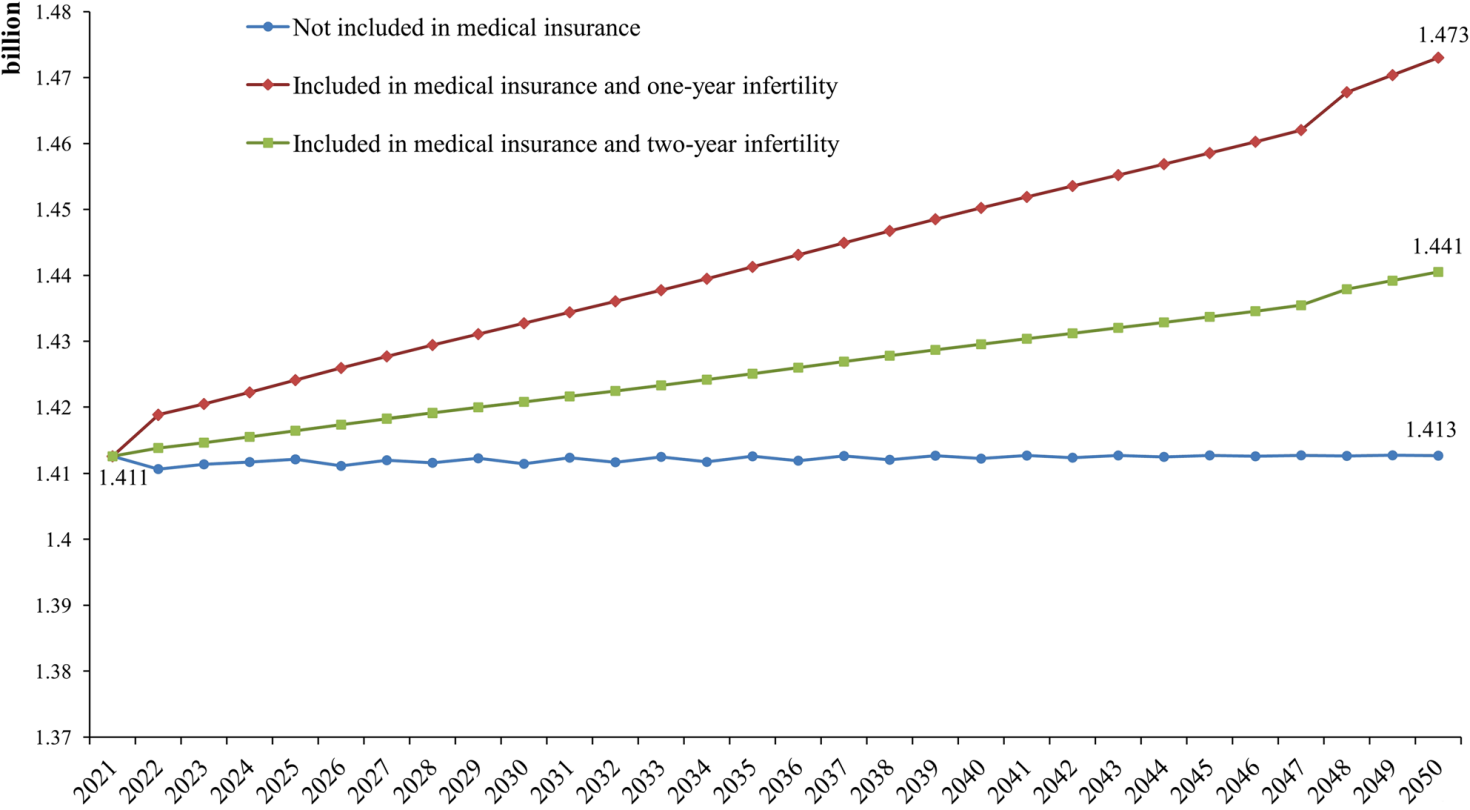
Population forecast from 2022 to 2050 based on the neural network model.

If ART is included in MI, 3.348–9.624 million live infants would be born in 2022, and 0.970–1.524 million newborns would be born in 2023, accounting for approximately 8–13% of the population born that year, and this proportion would be maintained thereafter. The childbearing age of the new population was assumed to be the current average childbearing age of 27 years^1^. We predict that the total population is expected to be 1.450 (2-year infertility) billion and 1.478 billion (1-year infertility) in 2050. If ART is not covered, the total population of mainland China will remain stable, reaching 1.413 billion in 2050. The population prediction results are shown in Fig. 3.

## Discussion

### China's population development trend

In recent years, China has been sliding into a "low fertility trap". From 1990 to 2021, China's birth rate dropped from 21.06 to 7.52‰, and the population growth rate dropped from 14.39 to 0.34‰. At the same time, the ageing rate of the population over the age of 65 years rose from 5.6 to 14.2%. Aware of the seriousness of this problem, the Chinese government has made several policy adjustments in the past decade to stimulate fertility. However, the effect of these policies seems to be limited. In 2021, there were 10.62 million births, with a birth rate of 7.52‰, with both being the lowest since 1978. The population crisis has become one of the hottest topics. At the National People's Congress (NPC) and the Chinese People's Political Consultative Conference (CPPCC) in 2022, a total of 9 proposals, the most ever, were aimed at raising the fertility rate.

### Infertility rate and number in China

At present, there is no unified opinion on the definition of infertility. In different studies, the denominator of the infertility rate is also inconsistent^5^. It was difficult for us to accurately estimate the number of infertile couples in China. Therefore, we tried to solve this problem through meta-analysis. The infertility rate of all couples was 0.015–0.042, and that of couples at risk was 0.089–0.153. A review reported that the one-year infertility rate of married and voluntary women aged 20–44 years is approximately 9% worldwide^20^, which seemed to be consistent with our conclusion. From the 1970s to the 1980s, the two-year infertility rate of newly married women in China was 6.89%^21^, which was lower than the current rate of 8.9%. We also found that the visit rate of infertile couples was approximately 40%, of which approximately 20% underwent ART, which was consistent with previous literature reports^2,10^. Our results indicate that infertility is mainly due to female factors. But men patients are easily missed detection. Due to social beliefs and childbearing expectations from the female, in most cases only infertility evaluations are conducted on women, without evaluating male partners^22^.

Based on the prevalence rate, we speculated that there were 4.102–11.792 million infertile couples in China, with an annual increase of 1.189–1.867 million. This was much lower than the 50 million previously reported in some studies^10,23,24^, which mistakenly multiplied the infertility rate of couples at risk by the number of all couples, thus seriously overestimating the number of people experiencing infertility. People who have given birth and do not want to give birth again, even if they have lost their reproductive function, should not be counted in the infertile population. According to the research of the Center for Population and Development Studies of Renmin University of China, the lifetime infertility rate of women over the age of 40 years was 0.27–1.00%, which was close to our results^24^.

### ART in China

After the first baby was born as a result of IVF in China in 1988, an increasing number of infertile couples has benefited from ART^23^. According to the information released by the National Health Commission, there are 536 ART institutions in China, of which 411 can carry out IVF and ICSI, and 78 can carry out PGD/PGS. There are more than 1 million ART cycles and more than 300,000 newborns every year^8^. The average LBR per transplantation in China is 40.5%, similar to that of 43% in the United States^25^. However, the multiple birth rate of ART in China is 25.4%, which is much higher than that of 13.1% in the United States^25^ and 0.65% of natural pregnancies in China^26^.

ART is a long and expensive process. In China, the cost of ART needs to be borne by the patients themselves. Economic research on ART has mainly been concentrated in Europe and the United States, accounting for 88% of studies^27^. Prior to this, there has been no multicentre survey, review or meta-analysis on the cost of ART in China. Our results showed that the cost per live birth was 67,002 yuan and that per complete cycle was 23,901 yuan. Age composition was an important factor affecting the cost. Among the people we included, 27.6% were women over the age of 35 years, which was in line with the actual proportion of 30%^13–15^, indicating that the costs were unbiased. Based on the clinical data of the reproductive medicine centre of Peking University People's Hospital in 2008, Dr. Zheng studied the economic burden of infertility diseases in China^10^. He estimated that the direct medical cost per patient was 62,150 yuan, which was close to our results. Compared with the average hospitalization expenses of 30 serious diseases listed in the 2020 China Health Statistics Yearbook (published by China Union Medical College Press in 2021), only the cost of coronary artery bypass grafting for myocardial infarction (74,942 yuan) was higher than the direct medical cost of infertility. Compared to countries worldwide, China's disease burden is high. And the probability of high-income groups receiving ART treatment is much higher than that of low-income groups^28^. At present, 44 countries have included all or part of the costs of ART in MI, mainly in the Americas and Western Europe. The covered costs include: drugs, ART procedures and hospital stays, complication management, and pregnancy diagnosis^29^. In the United States, the use rate of in IVF is 277% higher in states with complete insurance coverage than in states without coverage^30^.

### Financial expenses and cost-effectiveness after the inclusion of ART in MI

To verify the stability of the results, we used the average cost per complete cycle and per live birth to calculate the cost of ART for all infertile couples. The results were very close. We took the average of the two as our estimate. Finally, we found that the fund would need to pay 72.313–207.878 billion yuan, and the subsequent annual payment would be 20.961–32.913 billion yuan. The LEV per complete cycle was 3.174 million yuan; the financial input‒output ratio was 13.022.

Of course, it is impossible that 100% of infertile couples will choose to undergo ART. At present, this proportion is 38%^9^, and it may increase with the inclusion of ART in MI. Other treatments include artificial insemination, clomiphene, surgery, and observation, which together account for 62% of all treatments^9^, but only 10–20% of all treatment costs^10^. This means that when the proportion of ART decreases by 10%, the total cost will decrease by 8–9%. At present, ART accounts for approximately 40% of infertile couples, so the actual financial burden may be less than half of our previous estimate.

If MI does not cover ART, the total population of mainland China is expected to reach 1.413 billion in 2050, which is consistent with Yi Zeng's forecast of 1.420 billion^31^. With the inclusion of ART in MI, the total population would be expected to be 1.450–1.478 billion in 2050. This means that by 2050, 37–65 million people would be born due to the inclusion of ART in MI.

### Feasibility of MI including ART

Whether ART needs to be incorporated into MI is controversial. In March 2021, a deputy of the NPC suggested that infertility treatment be included in MI to improve population growth. The NHSA replied that ovulation-promoting drugs such as bromocriptine, triptorelin and clomiphene have been included in MI, and supported the inclusion of ART in commercial MI. However, in fact, these drugs can only be paid by MI in some special departments, while patients undergoing ART need to pay for these drugs themselves. Moreover, at present, less than 15% of resident families in China buy commercial MI^32^, and the vast majority of infertile couples cannot benefit from commercial MI. With the implementation of China's three-child policy, the support for the inclusion of ART in MI is increasing. In 2022, at least five NPC deputies proposed incorporating ART into MI. Starting from July 1, 2023, Beijing has included ART in medical insurance.

Incorporating ART into MI is very important^33^. First, the right to have children is considered a fundamental human right^34^, and the government has the responsibility and obligation to provide help for infertile couples. Second, the inclusion of ART in MI will indeed help alleviate the decline in the birth rate. Third, infertility will increase patients' depression and anxiety^35^ and lead to social problems such as divorce and domestic violence^36^. Fourth, twins cost four times as much as singletons, while triplets cost ten times as much as singletons^37^, and the inclusion of ART in MI can effectively reduce the incidence of multiple births^30,38^.

The income of China’s MI fund exceeds the expenditure every year. In 2021, the fund income was 2, 871.03 billion yuan, the expenditure was 2, 401.11 billion yuan, and the accumulated balance was 3, 612.15 billion yuan. The cost of incorporating ART into MI accounted for only 2–6% of the current fund balance; the subsequent annual expenses accounted for only 4–7% of the annual fund balance. This was assuming that all infertile couples would undergo ART, and the actual cost was likely to be lower. At least, from the data point of view, it seemed feasible to include ART in MI, and the benefits were obvious. It should be noted that about 30% of the surplus of the MI fund is deposited into the insured's personal account. But according to the above calculation, after deducting the surplus from personal accounts, the funds should also be sufficient. Lin Wang also believed that it is feasible to access selective reimbursement and subsidies for those in particular need ^12^.

How to incorporate ART into MI is also a topic worth exploring. Referring to the Affordable Care Act of the United States^39^, it should have three goals: provide ART healthcare for all patients, control costs of healthcare, and improve the quality of healthcare. Expand fund sources, training doctors, professional regulatory agencies, opposing waste, reasonable payment systems, reward and punishment measures are considered good experiences^39^. It is also important to adopt low-cost ART treatment strategies^40^ and implement strict admission policies^12^. In addition, ART coverage for MI by government can be launched in the beginning by partial reimbursement of ART treatment expenses for special groups such as patients belonging to low- or middle-income classes, and elderly patients. Finally, we can also determine the payment ratio based on the number of children; For those who have never given birth, the payment ratio is the highest, and as the number of children increases, the payment ratio gradually decreases.

## Conclusion

In this study, we calculated the financial costs and benefits if ART was included in MI and found that its inclusion was feasible. However, this study has some limitations. First, we tried our best to avoid using experiences or hypotheses in estimating the cost of ART and obtained data through meta-analysis or government bulletins to improve the accuracy of our research results, but there was still missing information. The assumption of some costs or situations may have affected the calculation of the disease burden. Second, we calculate the cost through the mathematical model rather than through investigation, but the real situation is much more complicated than our mathematical model. For example, the data we used for decision tree analysis, including success rate and cost, came from meta-analysis, but these values should be an interval rather than an exact point. Thirdly, this study did not consider that different income groups have different affordability for ART costs. Finally, there is no unified definition for infertility, ART success rate, and cost composition, and the adjustment of MI policy will lead to changes in the quantity and cost of ART, all of which increase the difficulty and error of calculation. The economic burden of infertility patients in China is high compared to countries worldwide. The Chinese government may be able to do more. We hope that our research results can provide some references for the decision-making of the Chinese government to help millions of infertile couples who are struggling and in pain.

### Supplementary Information

Supplementary Information 1.

Supplementary Information 2.

Supplementary Information 3.

Supplementary Information 4.

## Data Availability

The datasets used and/or analysed during the current study available from the corresponding author on reasonable request.

## Abbreviations

ART: Assisted reproductive technology
MI: Medical insurance
IVF: In vitro fertilization
ICSI: Intracytoplasmic sperm injection
PGD/PGS: Preimplantation genetic diagnosis
AI: Artificial insemination
LBR: Live birth rate
CLBR: Cumulative live birth rate
LEV: Lifelong economic value
GDP: Gross domestic product

## References

[1] He D, Zhang X, Zhuang Y, WangYang ZS (2018). China fertility status report, 2006–2016: An analysis based on 2017 China fertility survey. Popul. Res..

[2] Qiao J, Wang Y, Li X, Jiang F, Zhang Y, Ma J, Song Y, Fu W, Pang R, Zhu Z, Zhang J, Qian X, Wang L, Wu J, Chang HM, Leung PCK, Mao M, Ma D, Guo Y, Qiu J, Liu L, Wang H, Norman RJ, Lawn J, Black RE, Ronsmans C, Patton G, Zhu J, Song L, Hesketh T (2021). A Lancet Commission on 70 years of women's reproductive, maternal, newborn, child, and adolescent health in China. Lancet.

[3] Sauer MV (2015). Reproduction at an advanced maternal age and maternal health. Fertil. Steril..

[4] Levine H, Jørgensen N, Martino-Andrade A, Mendiola J, Weksler-Derri D, Mindlis I, Pinotti R, Swan SH (2017). Temporal trends in sperm count: a systematic review and meta-regression analysis. Hum. Reprod. Update.

[5] Gurunath S, Pandian Z, AndersonBhattacharya RAS (2011). Defining infertility—A systematic review of prevalence studies. Hum. Reprod. Update.

[6] Zhang P (2020). What can be learned from the Chinese experience in response to infertility. Eur. J. Contracept Reprod. Health Care.

[7] Luo Y, SuZheng BX (2021). Trends and challenges for population and health during population aging-China, 2015–2050. China CDC Wkly..

[8] Bai F, Wang DY, Fan YJ, Qiu J, Wang L, Dai Y, Song L (2020). Assisted reproductive technology service availability, efficacy and safety in mainland China: 2016. Hum. Reprod..

[9] Audibert C, Glass D (2015). A global perspective on assisted reproductive technology fertility treatment: An 8-country fertility specialist survey. Reprod. Biol. Endocrinol..

[10] Zheng X, Qiu Y (2012). Disease burden of infertility in China. Chin. J. Public Health.

[11] Chambers GM, Keller E, Choi S, Khalaf Y, Crawford S, Botha W, Ledger W (2020). Funding and public reporting strategies for reducing multiple pregnancy from fertility treatments. Fertil. Steril..

[12] Wang L, Zhu Y, Wang T, Xu X, Tang Q, Li J, Wang Y, Hu W, Wu W (2022). Feasibility analysis of incorporating infertility into medical insurance in China. Front. Endocrinol. (Lausanne).

[13] Hu L, Bu Z, Huang G, Sun H, DengSun CY (2020). Assisted reproductive technology in China: Results generated from data reporting system by CSRM from 2013 to 2016. Front. Endocrinol. (Lausanne).

[14] Yang J, Deng C, Huang X, Liu P, Zhou C, Feng Y, Hao G, Lu W, Quan S, Shen H (2020). Chinese society reproductive medicine annual report: Data analysis of ART in 2017. J. Reprod. Med..

[15] Yang J, Deng C, Huang X, Liu P, Zhou C, Feng Y, Hao G, Lu W, QuanShen SH (2021). Annual report on assisted reproductive technology of Chinese Society of Reproductive Medicine in 2018. J. Reprod. Med..

[16] Zegers-Hochschild F, Adamson GD, de Mouzon J, Ishihara O, Mansour R, Nygren K, Sullivan E, Vanderpoel S (2009). International Committee for Monitoring Assisted Reproductive Technology (ICMART) and the World Health Organization (WHO) revised glossary of ART terminology, 2009. Fertil. Steril..

[17] Pan W, Tu H, Jin L, Hu C, Li Y, Wang R, HuangLiao WS (2019). Decision analysis about the cost-effectiveness of different in vitro fertilization-embryo transfer protocol under considering governments, hospitals, and patient. Medicine (Baltimore).

[18] Connolly M, Gallo F, Hoorens S, Ledger W (2009). Assessing long-run economic benefits attributed to an IVF-conceived singleton based on projected lifetime net tax contributions in the UK. Hum. Reprod..

[19] Li HWR, Tank J, Haththotuwa R (2018). Updated status of assisted reproductive technology activities in the Asia-Oceania region. J. Obstet. Gynaecol. Res..

[20] Boivin J, Bunting L, CollinsNygren JAKG (2007). International estimates of infertility prevalence and treatment-seeking: Potential need and demand for infertility medical care. Hum. Reprod..

[21] Fang K, Deng X, Gao E (1992). Analysis of infertility rate of first married women in China from 1976 to 1985. Reprod. Contracept..

[22] Schlegel PN, Sigman M, Collura B, de Jonge CJ, Eisenberg ML, Lamb DJ, Mulhall JP, Niederberger C, Sandlow JI, Sokol RZ, Spandorfer SD, Tanrikut C, Treadwell JR, Oristaglio JT, Zini A (2021). Diagnosis and treatment of infertility in men: AUA/ASRM guideline part I. Fertil. Steril..

[23] Qiao J, Feng HL (2014). Assisted reproductive technology in China: Compliance and non-compliance. Transl. Pediatr..

[24] Zhai Z, Liu W (2020). Estimating the prevalence of infertility in China using census data. Popul. Res..

[25] Sunderam S, Kissin DM, Zhang Y, Jewett A, Boulet SL, Warner L, Kroelinger CD, Barfield WD (2020). Assisted reproductive technology surveillance—United States, 2017. MMWR Surveill. Summ..

[26] Lu X, Zhang J, Liu Y, Wang T, LuLi YX (2013). Epidemiology of twin births in southeast China: 1993–2005. Twin Res. Hum. Genet..

[27] Moolenaar LM, Vijgen SM, Hompes P, van der Veen F, Mol BW, Opmeer BC (2013). Economic evaluation studies in reproductive medicine: A systematic review of methodologic quality. Fertil. Steril..

[28] Brautsch LAS, Voss I, Schmidt L, Vassard D (2023). Social disparities in the use of ART treatment: A national register-based cross-sectional study among women in Denmark. Hum. Reprod..

[29] Barriere P, Porcu-Buisson G, Hamamah S (2018). Cost-effectiveness analysis of the gonadotropin treatments HP-hMG and rFSH for assisted reproductive technology in France: A Markov model analysis. Appl. Health Econ. Health Policy.

[30] Peipert BJ, Montoya MN, Bedrick BS, Seifer DB, Jain T (2022). Impact of in vitro fertilization state mandates for third party insurance coverage in the United States: A review and critical assessment. Reprod. Biol. Endocrinol..

[31] Zeng Y, Hesketh T (2016). The effects of China's universal two-child policy. Lancet.

[32] Wang S, LiuGuo AW (2021). Public and commercial medical insurance enrollment rates of rural-to-urban migrants in China. Front. Public Health.

[33] Afferri A, Allen H, Booth A, Dierickx S, Pacey A, Balen J (2022). Barriers and facilitators for the inclusion of fertility care in reproductive health policies in Africa: A qualitative evidence synthesis. Hum. Reprod. Update.

[34] Inhorn MC, Patrizio P (2015). Infertility around the globe: new thinking on gender, reproductive technologies and global movements in the 21st century. Hum. Reprod. Update.

[35] Gdańska P, Drozdowicz-Jastrzębska E, Grzechocińska B, Radziwon-Zaleska M, WęgrzynWielgoś PM (2017). Anxiety and depression in women undergoing infertility treatment. Ginekol. Pol..

[36] Stellar C, Garcia-Moreno C, Temmerman M, van der Poel S (2016). A systematic review and narrative report of the relationship between infertility, subfertility, and intimate partner violence. Int. J. Gynaecol. Obstet..

[37] Collins J (2007). Cost efficiency of reducing multiple births. Reprod. Biomed. Online.

[38] Provost MP, Thomas SM, Yeh JS, Hurd WW, Eaton JL (2016). State insurance mandates and multiple birth rates after in vitro fertilization. Obstet. Gynecol..

[39] Adkinson JM, Chung KC (2014). The patient protection and Affordable Care Act: A primer for hand surgeons. Hand Clin..

[40] Chiware TM, Vermeulen N, Blondeel K, Farquharson R, Kiarie J, Lundin K, Matsaseng TC, Ombelet W, Toskin I (2021). IVF and other ART in low- and middle-income countries: A systematic landscape analysis. Hum. Reprod. Update.

