# Supplementary material for: Feasibility analysis of China's medical insurance coverage of assisted reproductive technology
Source: Sci Rep. 2024 Apr 5;14:7998. doi: 10.1038/s41598-024-58640-4 (PMC10997767; doi:10.1038/s41598-024-58640-4)
Supplement: Supplementary file 1 — Supplementary Information 1. [file 41598_2024_58640_MOESM1_ESM.docx]

**Supplementary material 1**

**Details of the literature search**

| **Item** | **Content** |
| --- | --- |
| **Search strategies** | We collected the data of this study from Chinese and English language sources. English literatures were searched on PubMed, Cochrane and EMBASE. Chinese sources came from WanFang search engine, Chinese National Knowledge Infrastructure (CNKI) search engine and VIP search engine. We also manually searched references in key articles by hand. The search time was from January 2000 to January 2022. Two reviewers independently conducted literature screening, quality evaluation and data extraction, and they proofread each other’s work. In case of dispute, the third reviewer participated in the discussion and finally reached an agreement. All the literatures were officially published and full-text literatures were available. For questions in the article, we contacted the author by email. |
| **Search term** | Topic A (Prevalence of infertility in China): We searched the literature on infertility rates. The following search terms were used: (China or Chinese) and (infertility or sterility or infecundity or childless) and (prevalence or epidemiology or rate or incidence).  Topic B (Success rate of ART in China): We searched the literature on the live birth rate of different oocyte retrieval cycles and different transfer cycles in first oocyte retrieval. The following search terms were used: (assisted reproduction or IVF or vitro fertilization or embryo transfer) and ((cumulative live birth rate or CLBR) or (cumulative and (live birth rate or LBR or persistent pregnancy rate))) and (China or Chinese) .  Topic C (Cost per live birth of ART in China): We searched the literature on the cost of each live birth rate or persistent pregnancy in China. The following search terms were used: (assisted reproduction or IVF or vitro fertilization or embryo transfer) and (cost or economic or benefit) and (live birth rate or LBR or persistent pregnancy rate) and (China or Chinese).  Topic D (Cost per live birth of ART in various countries): We searched the literature on the cost of each live birth rate or persistent pregnancy. The following search terms were used: (assisted reproduction or IVF or vitro fertilization or embryo transfer) and (cost or economic or benefit) and (live birth rate or LBR or persistent pregnancy rate). |
| **Quality evaluation** | We used the quality evaluation tool of The Joanna Briggs Institute (JBI) for prevalence research to evaluate the literature quality of topic A^[1](#_ENREF_1)^. The item scores range between 0 and 9, 1 point for each item, and less than or equal to 5 points means low quality. Limited by the type of research, we did not evaluate the quality of Topic B, Topic C and Topic D. |
| **Study selection** | Topic A: Inclusion criteria: ① The wives were between 20~49 years old, and were from Chinese mainland, excluding Taiwan, Hongkong and Macao. ② The survey method was sampling survey or general survey. ③ The sample size was more than 1000. Exclusion criteria: ① The same data were reported in multiple articles. ② Case reports or reviews. ③ Non random sampling or non representative population. ④ Studies with unclear definitions of infertility. ⑤ Quality score ≤ 5.  Topic B: Inclusion criteria: ① All patients in a hospital for a certain period of time. ② The number of participants and live births in each cycle can be obtained. ③ The sample size was more than 1000. Exclusion criteria: ① The same data were reported in multiple articles. ② Case reports or reviews. ③ The sample was a selective special population.  Topic C: Inclusion criteria: ① Data such as sample size and cost per live birth can be obtained. ② Expenses refer to the direct medical expenses after initiated cycles, including operation expenses, medical expenses, examination expenses, etc., but excluding the physical examination costs before starting, complication treatment costs, pregnancy and childbirth costs, as well as food, lost wages, traffic, accommodation and other indirect costs. Exclusion criteria: ① The same data were reported in multiple articles. ② Case reports or reviews. ③ Specific groups with significantly higher costs, such as repeated cycles after multiple failures.  Topic D: Inclusion criteria: ① The cost of per live birth can be found directly or calculated indirectly. ② Expenses refer to the direct medical expenses after initiated cycles, including operation expenses, medical expenses, examination expenses, etc., but excluding the physical examination costs before starting, complication treatment costs, pregnancy and childbirth costs, as well as work delay, traffic, accommodation and other indirect costs. Exclusion criteria: ① The same data were reported in multiple articles. ② Specific groups with significantly higher costs, such as older women ≥ 35 years old, oocyte freezing and PGD. |
| **Data extraction** | Topic A: author, investigation time, sample size, number of infertile couples, diagnostic criteria (one year or two years), population (entire couples or risk couples), age, treatment data and sampling method.  Topic B: author, investigation time, sample size, number of live births and cycle type.  Topic C: author, investigation time, sample size, treatment method, direct medical expenses and age.  Topic D: author, investigation time, sample size, direct medical expenses, country, cost per live birth and currency. |

**References**

1. Munn Z, Moola S, Lisy K, Riitano D, Tufanaru C. Methodological guidance for systematic reviews of observational epidemiological studies reporting prevalence and cumulative incidence data. Int J Evid Based Healthc. 2015; **13**(3): 147-53.
