# Supplementary figures and images for: Feasibility analysis of China's medical insurance coverage of assisted reproductive technology
Source: Sci Rep. 2024 Apr 5;14:7998. doi: 10.1038/s41598-024-58640-4 (PMC10997767; doi:10.1038/s41598-024-58640-4)

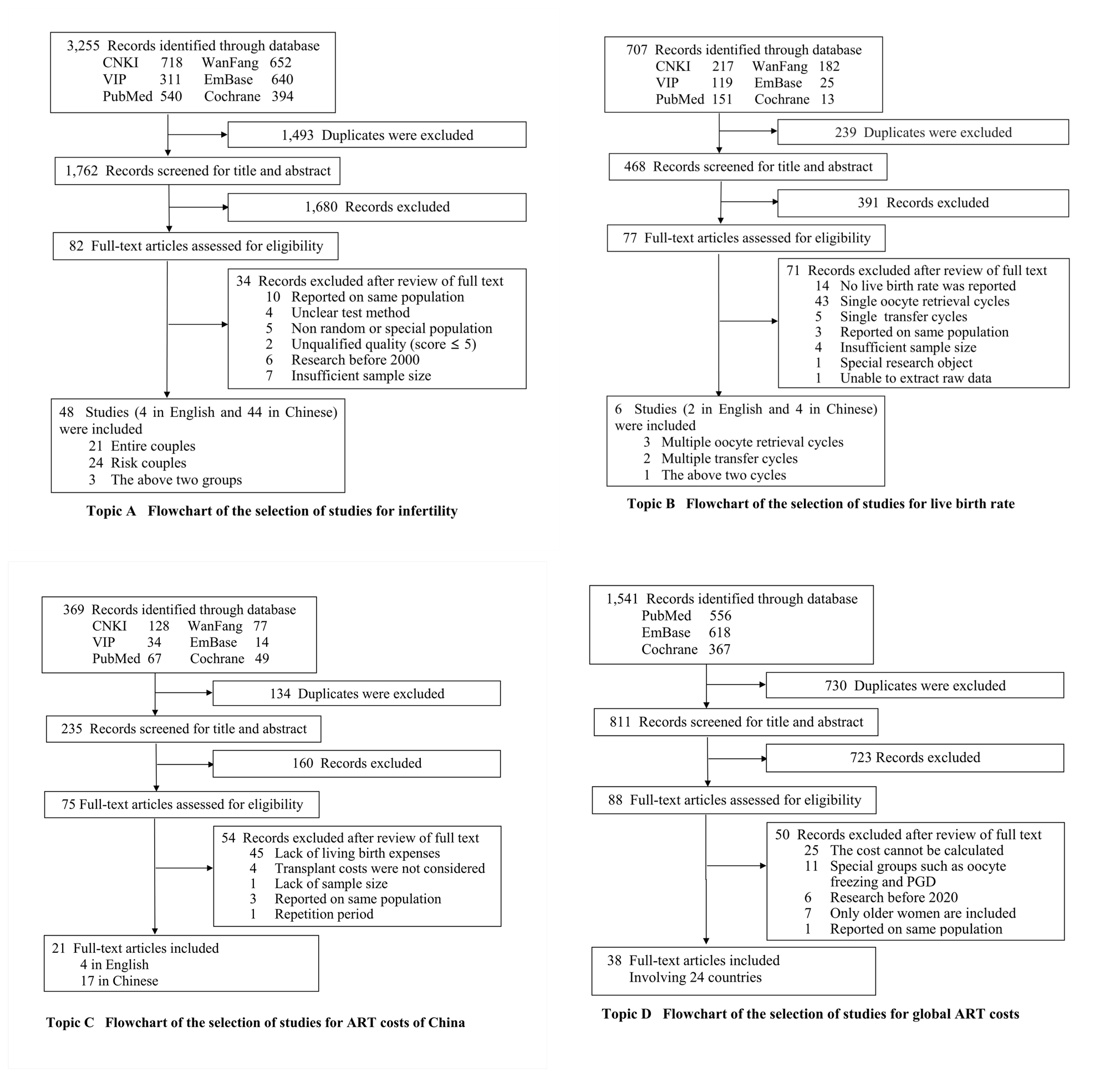

Supplement: Supplementary file 2 — Supplementary Information 2. [file 41598_2024_58640_MOESM2_ESM.jpg]
