# Supplementary material for: Feasibility analysis of China's medical insurance coverage of assisted reproductive technology
Source: Sci Rep. 2024 Apr 5;14:7998. doi: 10.1038/s41598-024-58640-4 (PMC10997767; doi:10.1038/s41598-024-58640-4)
Supplement: Supplementary file 3 — Supplementary Information 3. [file 41598_2024_58640_MOESM3_ESM.docx]

**Supplementary material 3**

**Information on the literature included in the study**

| **Topic A Literature included in meta-analysis of infertility rate in China** | | | | | | | | | |
| --- | --- | --- | --- | --- | --- | --- | --- | --- | --- |
| **ID** | **First author name** | **Investigation time** | **Sample size** | **Group** | **Diagnostic criteria** | **Age grouping** | **Treatment data** | **Sampling method** | **Quality score** |
| 1 | Liu D^[1](#_ENREF_1)^ | 2015 | 4,697 | Entire couples | One year | No | No | Sampling survey | 7 |
| 2 | Chen H^[2](#_ENREF_2)^ | 2005 | 2,316,348 | Entire couples | One year | No | Yes | General survey | 7 |
| 3 | Yang Y^[3](#_ENREF_3)^ | 2007~2008 | 5,632 | Entire couples | Two years | Yes | No | Sampling survey | 8 |
| 4 | Guo X^[4](#_ENREF_4)^ | 2001~2005 | 5,325,844 | Entire couples | One year and two years | No | No | General survey | 7 |
| 5 | Zhang J^[5](#_ENREF_5)^ | 2007~2009 | 11,946 | Entire couples | Two years | Yes | No | Sampling survey | 6 |
| 6 | Xu A^[6](#_ENREF_6)^ | 2018 | 1,551 | The two groups | One year | Yes | No | Sampling survey | 7 |
| 7 | Yu F^[7](#_ENREF_7)^ | 2014 | 3,376 | Entire couples | One year | Yes | Yes | Sampling survey | 7 |
| 8 | Liu Z^[8](#_ENREF_8)^ | 2008~2009 | 10,500 | Entire couples | One year | Yes | No | Sampling survey | 8 |
| 9 | Wang J^[9](#_ENREF_9)^ | 2004 | 6,860 | Entire couples | One year | No | No | Sampling survey | 6 |
| 10 | Tang L^[10](#_ENREF_10)^ | 2002~2003 | 19,595 | Risk couples | One year | Yes | No | General survey | 9 |
| 11 | Qu S^[11](#_ENREF_11)^ | 2016 | 7,294 | Risk couples | One year | Yes | No | General survey | 8 |
| 12 | Huang J^[12](#_ENREF_12)^ | 2007 | 18,893 | Risk couples | One year | Yes | No | General survey | 9 |
| 13 | Chen D^[13](#_ENREF_13)^ | 2012 | 21,239 | Entire couples | One year | Yes | No | Sampling survey | 7 |
| 14 | Jian N^[14](#_ENREF_14)^ | 2003 | 100,842 | Entire couples | Two years | No | Yes | Sampling survey | 7 |
| 15 | Zhang L^[15](#_ENREF_15)^ | 2012 | 19,588 | Risk couples | One year | No | Yes | General survey | 6 |
| 16 | Ni S^[16](#_ENREF_16)^ | 2008 | 3,847 | Entire couples | One year | Yes | Yes | Sampling survey | 7 |
| 17 | Huang Q^[17](#_ENREF_17)^ | 2007 | 2,867 | Risk couples | One year | Yes | Yes | General survey | 9 |
| 18 | Tian L^[18](#_ENREF_18)^ | 2006 | 571,741 | Entire couples | Two years | Yes | No | Sampling survey | 8 |
| 19 | Yang H^[19](#_ENREF_19)^ | 2012 | 4,394 | Entire couples | One year | No | No | Sampling survey | 8 |
| 20 | Cao Y^[20](#_ENREF_20)^ | 2011 | 3,175 | Risk couples | One year | Yes | No | Sampling survey | 8 |
| 21 | Jiang Y^[21](#_ENREF_21)^ | 2012 | 2,326 | Entire couples | One year | Yes | No | Sampling survey | 8 |
| 22 | Yan Y^[22](#_ENREF_22)^ | 2017 | 4,776 | Risk couples | One year | Yes | No | General survey | 8 |
| 23 | Wang Y^[23](#_ENREF_23)^ | 2000 | 210,650 | Entire couples | One year | No | Yes | General survey | 7 |
| 24 | Wang Y^[24](#_ENREF_24)^ | 2000 | 93,119 | Entire couples | One year | No | No | General survey | 7 |
| 25 | Hou Q^[25](#_ENREF_25)^ | 2004 | 5,468 | Entire couples | One year | Yes | No | Sampling survey | 8 |
| 26 | Cai X^[26](#_ENREF_26)^ | 2008~2009 | 1,835 | Risk couples | One year | No | No | Sampling survey | 7 |
| 27 | Zhang Y^[27](#_ENREF_27)^ | 2010~2015 | 1,997 | Entire couples | One year | Yes | No | Sampling survey | 6 |
| 28 | Xu M^[28](#_ENREF_28)^ | 2015 | 7,559 | Risk couples | One year and two years | No | No | Sampling survey | 6 |
| 29 | Zhang Z^[29](#_ENREF_29)^ | 1998~2001 | 32,637 | Risk couples | One year and two years | No | Yes | General survey | 7 |
| 30 | Ou H^[30](#_ENREF_30)^ | 2015~2017 | 1,842 | Risk couples | One year | Yes | No | General survey | 6 |
| 31 | Gao J^[31](#_ENREF_31)^ | 2001 | 28,511 | Risk couples | One year | Yes | No | Sampling survey | 9 |
| 32 | Yang Y^[32](#_ENREF_32)^ | 2013 | 4,752 | Risk couples | One year and two years | No | Yes | General survey | 9 |
| 33 | Xing Z^[33](#_ENREF_33)^ | 2016 | 33,685 | Risk couples | One year and two years | Yes | No | Sampling survey | 7 |
| 34 | Li Y^[34](#_ENREF_34" \o "Li, 2021(in Chinese) #164)^ | 2019 | 1,494 | The two groups | One year | Yes | No | Sampling survey | 9 |
| 35 | Wang B^[35](#_ENREF_35" \o "Wang, 2019(in Chinese) #166)^ | 2017~2019 | 9,320 | Risk couples | One year | Yes | No | Sampling survey | 7 |
| 36 | Hou L^[36](#_ENREF_36" \o "Hou, 2011(in Chinese) #167)^ | 2005~2006 | 52,870 | Risk couples | Two years | Yes | Yes | Sampling survey | 9 |
| 37 | Feng Y^[37](#_ENREF_37" \o "Feng, 2017(in Chinese) #165)^ | 2014~2015 | 1,953 | Entire couples | One year | No | No | Sampling survey | 8 |
| 38 | Cong J^[38](#_ENREF_38)^ | 2014 | 4,232 | Risk couples | One year | No | No | Sampling survey | 8 |
| 39 | Chen J^[39](#_ENREF_39)^ | 2011~2012 | 6,904 | Risk couples | One year | Yes | No | General survey | 8 |
| 40 | Zhou Z^[40](#_ENREF_40)^ | 2010~2011 | 17,275 | Risk couples | One year | Yes | Yes | Sampling survey | 9 |
| 41 | Ye Y^[41](#_ENREF_41)^ | 2016 | 5,536 | Risk couples | One year | Yes | No | Sampling survey | 7 |
| 42 | Liu S^[42](#_ENREF_42)^ | 2008~2009 | 8,935 | Risk couples | One year and two years | No | Yes | Sampling survey | 6 |
| 43 | An M^[43](#_ENREF_43)^ | 2008 | 13,612 | The two groups | One year and two years | No | No | General survey | 6 |
| 44 | Zhang Z^[44](#_ENREF_44)^ | 2001~2017 | 1,240 | Entire couples | One year | No | No | Sampling survey | 8 |
| 45 | Jia Y^[45](#_ENREF_45)^ | 2000~2004 | 5,000 | Risk couples | Two years | No | Yes | Sampling survey | 7 |
| 46 | Meng Q^[46](#_ENREF_46)^ | 2009~2012 | 1,627 | Risk couples | One year and two years | Yes | No | General survey | 7 |
| 47 | Wei T^[47](#_ENREF_47)^ | 2018 | 9,332 | Risk couples | One year | No | No | Sampling survey | 7 |
| 48 | Liu J^[48](#_ENREF_48)^ | 2016~2019 | 2,332 | Entire couples | One year | Yes | No | Sampling survey | 7 |
| **Topic B The literature included in the meta-analysis of the success rate of ART in China** | | | | | | | | | |
| **ID** | **First author name** | **Investigation time** | **Sample size** | **Cycle type** | **—** | **—** | **—** | **—** | **—** |
| 49 | Chen L^[49](#_ENREF_49)^ | 2009~2015 | 42,661 | Multiple oocyte retrieval cycles | — | — | — | — | — |
| 50 | Hao, M^[50](#_ENREF_50" \o "Hao, 2016(in Chinese) #171)^ | 2011~2014 | 1,735 | The two cycles | — | — | — | — | — |
| 51 | Xu X^[51](#_ENREF_51)^ | 2016~2019 | 25,490 | Multiple oocyte retrieval cycles | — | — | — | — | — |
| 52 | Yuan L^[52](#_ENREF_52" \o "Yuan, 2016(in Chinese) #170)^ | 2007~2011 | 5,246 | Multiple oocyte retrieval cycles | — | — | — | — | — |
| 53 | Yang J^[53](#_ENREF_53)^ | 2016–2019 | 18,872 | Multiple transfer cycles | — | — | — | — | — |
| 54 | Huang L^[54](#_ENREF_54)^ | 2013~2016 | 14,311 | Multiple transfer cycles | — | — | — | — | — |
| **Topic C Literature included in meta-analysis of ART costs in China** | | | | | | | | | |
| **ID** | **First author name** | **Investigation time** | **Sample size** | **Treatment** | **Age grouping** | **—** | **—** | **—** | **—** |
| 55 | Li Y^[55](#_ENREF_55)^ | 2015~2019 | 1,622 | GnRH-a | Yes | — | — | — | — |
| 56 | Jing M^[56](#_ENREF_56)^ | 2015~2017 | 2,259 | GnRH-ant, GnRH-a | No | — | — | — | — |
| 57 | Wei X^[57](#_ENREF_57)^ | 2015~2017 | 120 | GnRH-ant, minimal-stimulation | Yes | — | — | — | — |
| 58 | Pan W^[58](#_ENREF_58)^ | 2014~2017 | 11,537 | GnRH-ant, GnRH-a | Yes | — | — | — | — |
| 59 | Zhang Y^[59](#_ENREF_59)^ | 2014~2016 | 302 | GnRH-a, GnRH-ant, minimal-stimulation, Others | Yes | — | — | — | — |
| 60 | Zhu C^[60](#_ENREF_60" \o "Zhu, 2021(in Chinese) #175)^ | 2018~2019 | 280 | GnRH-a, GnRH-ant | Yes | — | — | — | — |
| 61 | Zheng J^[61](#_ENREF_61)^ | 2017~2018 | 285 | GnRH-ant, PPOS, minimal-stimulation | Yes | — | — | — | — |
| 62 | Li J^[62](#_ENREF_62)^ | 2016 | 121 | GnRH-a | No | — | — | — | — |
| 63 | Xiao Y^[63](#_ENREF_63" \o "Yalin, 2016(in Chinese) #178)^ | 2009~2014 | 312 | GnRH-a | No | — | — | — | — |
| 64 | Dai F^[64](#_ENREF_64)^ | 2017~2019 | 575 | GnRH-a, GnRH-ant | Yes | — | — | — | — |
| 65 | Dai F^[65](#_ENREF_65)^ | 2015~2018 | 431 | GnRH-a, Others | Yes | — | — | — | — |
| 66 | Liu Y^[66](#_ENREF_66)^ | 2014~2015 | 1,400 | GnRH-a | No | — | — | — | — |
| 67 | Li Z^[67](#_ENREF_67" \o "Li, 2019(in Chinese) #182)^ | 2016~2018 | 78 | GnRH-a | No | — | — | — | — |
| 68 | Wu Y^[68](#_ENREF_68)^ | 2015~2021 | 158 | GnRH-a | Yes | — | — | — | — |
| 69 | Ni H^[69](#_ENREF_69" \o "Hao, 2011(in Chinese) #183)^ | 2007~2010 | 173 | GnRH-a, GnRH-ant | Yes | — | — | — | — |
| 70 | Sun Y^[70](#_ENREF_70)^ | 2006 | 101 | GnRH-a, GnRH-ant | No | — | — | — | — |
| 71 | Ji J^[71](#_ENREF_71)^ | 2008~2009 | 86 | GnRH-a, minimal-stimulation | No | — | — | — | — |
| 72 | Zhang R^[72](#_ENREF_72)^ | 2017~2018 | 282 | GnRH-ant | Yes | — | — | — | — |
| 73 | LV Z^[73](#_ENREF_73" \o "LV, 2017(in Chinese) #187)^ | 2016~2019 | 375 | minimal-stimulation, PPOS | No | — | — | — | — |
| 74 | Liu Y^[74](#_ENREF_74)^ | 2010~2018 | 699 | GnRH-a, Natural-cycle | Yes | — | — | — | — |
| 75 | Lin L^[75](#_ENREF_75)^ | 2017~2019 | 371 | GnRH-ant, minimal-stimulation, PPOS | Yes | — | — | — | — |
| **Topic D The literature included in the study of ART costs in various countries** | | | | | | | | | |
| **ID** | **First author name** | **Investigation time** | **Sample size** | **Country** | **Cost per live birth** | **Currency** | **—** | **—** | **—** |
| 76 | Pham CT^[76](#_ENREF_76)^ | 2013 | 8,781 | Australia | 28,775 | Dollar | — | — | — |
| 77 | Chambers GM^[77](#_ENREF_77)^ | 2003~2005 | — | Australia | 20,379 | Dollar | — | — | — |
| 78 | Chambers GM^[78](#_ENREF_78)^ | 2002 | — | Australia | 32,903 | Dollar | — | — | — |
| 79 | Fragoulakis V^[79](#_ENREF_79)^ | 2013 | — | Greece | 17,015 | Euro | — | — | — |
| 80 | Maldonado LG^[80](#_ENREF_80)^ | 2013 | 96 | Brazil | 17,386 | Dollar | — | — | — |
| 81 | De Sutter P^[81](#_ENREF_81)^ | 2002 | 3,300 | Belgium | 11,298 | Euro | — | — | — |
| 82 | Strandell A^[82](#_ENREF_82)^ | 2005 | 192 | Iceland, Denmark, Norway, Sweden | 21,324 | Dollar | — | — | — |
| 83 | Xue W^[83](#_ENREF_83)^ | 2016 | — | Germany | 12,159 | Euro | — | — | — |
| 84 | Gizzo S^[84](#_ENREF_84)^ | 2017 | — | Germany, Italy, Spain | Germany8,637, Italy9,112, Spain16,216 | Euro | — | — | — |
| 85 | Ferrier C^[85](#_ENREF_85)^ | 2004~2015 | 163 | France | 18,235 | Euro | — | — | — |
| 86 | Barriere P^[86](#_ENREF_86)^ | 2017 | — | France | 17,530 | Euro | — | — | — |
| 87 | Veleva Z^[87](#_ENREF_87)^ | 1995~2004 | 1,510 | Finland | 12,328 | Euro | — | — | — |
| 88 | Chambers GM^[88](#_ENREF_88)^ | 2009 | — | USA, Canada, UK, Scandinavia, Japan, Australia | USA41,132, Canada33,183, UK40,364,  Scandinavia24,485, Japan24,329, Australia25,843 | Dollar | — | — | — |
| 89 | Lukassen HG^[89](#_ENREF_89)^ | 2001~2003 | 107 | Netherlands | 9,128 | Euro | — | — | — |
| 90 | Groen H^[90](#_ENREF_90)^ | 2005~2008 | 891 | Netherlands | 17,714 | Euro | — | — | — |
| 91 | Fragoulakis V^[91](#_ENREF_91)^ | 2013 | — | Netherlands | 16,797 | Euro | — | — | — |
| 92 | van Tilborg TC^[92](#_ENREF_92)^ | 2017 | 1,515 | Netherlands | 8,875 | Euro | — | — | — |
| 93 | Polinder S^[93](#_ENREF_93)^ | 2002~2004 | 404 | Netherlands | 7,945 | Euro | — | — | — |
| 94 | Vélez MP^[94](#_ENREF_94)^ | 2009~2011 | 7,364 | Canada | 18,727 | Dollar | — | — | — |
| 95 | Vitek WS^[95](#_ENREF_95)^ | 2006~2010 | — | USA | 35,676 | Dollar | — | — | — |
| 96 | Murugappan G^[96](#_ENREF_96)^ | 2015 | 232 | USA | 45,300 | Dollar | — | — | — |
| 97 | Kansal-Kalra S^[97](#_ENREF_97)^ | 2005 | — | USA | 35,714 | Dollar | — | — | — |
| 98 | Lee M^[98](#_ENREF_98)^ | 2014~2016 | 132,961 | USA | 27,458 | Dollar | — | — | — |
| 99 | Maeda E^[99](#_ENREF_99)^ | 2010 | 155,916 | Japan | 1,974,000 | Yen | — | — | — |
| 100 | Almaslami F^[100](#_ENREF_100)^ | 2019 | 405 | Saudi Arabia | 36,258 | Saudi Riyal | — | — | — |
| 101 | Rafique M^[101](#_ENREF_101)^ | 2017~2018 | 826 | Saudi Arabia | 71,754 | Saudi Riyal | — | — | — |
| 102 | Yildiz MS^[102](#_ENREF_102)^ | 2016 | — | Turkey | 6,631 | Dollar | — | — | — |
| 103 | Hernandez Torres E^[103](#_ENREF_103)^ | 2012~2012 | 121 | Spain | 8,977 | Euro | — | — | — |
| 104 | Papaleo E^[104](#_ENREF_104)^ | 2012~2013 | 252 | Italy | 16,093 | Euro | — | — | — |
| 105 | Levi-Setti PE^[105](#_ENREF_105)^ | 2018 | 11,221 | Italy | 23,447 | Euro | — | — | — |
| 106 | Wechowski J^[106](#_ENREF_106)^ | 2008 | 986 | Britain | 11,857 | Pound | — | — | — |
| 107 | Maheshwari A^[107](#_ENREF_107)^ | 1997~2006 | 1,756 | Britain | 10,941 | Pound | — | — | — |
| 108 | Braam SC^[108](#_ENREF_108)^ | 2015~2017 | 311 | Vietnam | 4,084 | Dollar | — | — | — |
| 109 | Le KD^[109](#_ENREF_109)^ | 2015~2016 | 782 | Vietnam | 3,262 | Dollar | — | — | — |
| 110 | Khoa LD^[110](#_ENREF_110)^ | 2015~2016 | 394 | Vietnam | 10,248 | Dollar | — | — | — |
| 111 | Widge A^[111](#_ENREF_111)^ | 2005 | — | India | 2,667 | Dollar | — | — | — |
| 112 | Sini I^[112](#_ENREF_112)^ | 2016~2018 | 113 | Indonesia | 13,267 | Dollar | — | — | — |
| 113 | Saraeva NV^[113](#_ENREF_113)^ | 2019 | — | Russia | 7,500 | Dollar | — | — | — |

**References**

1. Liu D, Huang G, Ye H, Wen H. Survey of infertility rate in Chongqing couples with women aged20－45. Chongqing Medical Journal. 2009; **38**(34): 3089-91(in Chinese).

2. Chen H, He Y, Lu h, Tian K, Liu H, Wang Y. Investigation and Analysis on the basic situation of infertile couples in Chongqing. Chin J Fam Plann. 2008; **16**(4): 215-8(in Chinese).

3. Yang Y, Shen H, Chen J, Chen Z. A prevalence survey of infertility in Beijing, China. Natl Med J Chin. 2011; **91**(5): 313-6(in Chinese).

4. Guo X, Wang Y, Hao Q, Li X, Qi G, Li Y, et al. Prevalence of Infertility in Rural Areas of Shanxi Province. Chin J Fam Plann. 2006; **14**(6): 358-9(in Chinese).

5. Zhang J, Shan W, Li Q. Survey on the prevalence of infertility among married women of childbearing age in Chengde. Maternal and Child Health Care of China. 2010; **25**(3): 4881-3(in Chinese).

6. Xu A, Zhang R, Zhang L, Zhu L. Status investigation and analysis of influencing factors of infertility in Dali city. China Modern Doctor. 2020; **58**(26): 1-3(in Chinese).

7. Yu F, Luo s, Chen Y, Wu C. Investigation and Analysis on the prevalence of infertility and related conditions in Foshan City. Chinese Journal of Women and Children Health. 2015; **6**(2): 49-51(in Chinese).

8. Liu Z, Qiu J, Wang Y, Ni Y, Yang J. Prevalence of infertility among married women at reproductive age in Gansu Province. Chin J Fam Plann. 2011; **19**(9): 546-9(in Chinese).

9. Wang J, Song YJ, Zou X, Xu M. Etiology and epidemiology of infertility in Ganzhou City. Journal of Gannan Medical University. 2006; **26**(2): 168-71(in Chinese).

10. Tang L, Wang Q, Wen R, AI L, Huang J, Ma C, et al. A Cross-sectional Study on the Incidence of Infertility in Guangdong Province. Chin Prev Med. 2005; **6**(2): 106-8(in Chinese).

11. Qu S, Xu F, Huang X, Guan J. Epidemiological investigation on infertility among couples of childbearing age at first marriage in Yangjiang District, Guangdong Province. Guangdong Medical Journal. 2017; **38**(10): 1586-8(in Chinese).

12. Huang J, Tang Y, Wang Q, Wen R, Tang L, Liang G, et al. Incidence of infertility and its influencing factors among married residents in Guangdong province. Chin J Public Health Mar. 2013; **29**(2): 194-8(in Chinese).

13. Chen D, Huang T, Luo S, Wang K, Liang F, Feng Z. Investigation of the hazardous factors leading to female infertility in Beiliu city of Guangxi Province. Youjiang Medical Journal 2013; **41**(1): 13-5(in Chinese).

14. Jane N, Li Y, Feng L, Xin L, Zhong C. Epidemiological study on primary infertility in rural areas of Guizhou Province. Chin J Fam Plann. 2010; **18**(1): 33-5(in Chinese).

15. Zhang L, Liu J, Zhang Y, Li G, Wang J, Gao Z, et al. Prevalence of infertility in rural areas of Hebei Province. Chin J Fam Plann. 2013; **21**(4): 242-4(in Chinese).

16. Ni S, Huang J, Li S, Wu Z. Investigation and Analysis of Prevalence of Infertility in Dongguan City. Medicine and Society. 2010; **23**(8): 4-7(in Chinese).

17. Huang Q, Xu J, Huang B, Huang J, Wang Q, Hong Y, et al. Investigation on the incidence rate of infertility among newly married people in Heyuan. Guangdong Medical Journal. 2011; **32**(5): 646-7(in Chinese).

18. Tian L, Xu W, Yang J, Mao Z, Yin X. Survey on the prevalence of infertility in rural couples of childbearing age in Hubei Province. Chinese Journal of Health Statistics. 2008; **25**(6): 592-3(in Chinese).

19. Yang H, Ge C, Jiang F, Li J, Shen W. Epidemiological investigation on the prevalence of infertility among women of childbearing age in Huaihua, Hunan Province. Chin J Fam Plann. 2014; **22**(7): 475-7(in Chinese).

20. Yunfei C. An epidemiological survey of infertility for childbearing couple in Jiaxing city. China Modern Doctor. 2012; **50**(10): 25-7(in Chinese).

21. Yanping J. Investigation on the prevalence and risk factors of female infertility in Yinzhou District of Ningbo City. Modem Practical Medicine. 2012; **24**(6): 668-9(in Chinese).

22. Yan Y. Prevalence and risk factors of infertility in women of childbearing age in Qin Huangdao city. Chinese Journal of Birth Health & Heredity. 2019; **27**(4): 460-9(in Chinese).

23. Wang Y, Zhang Z, Zhang G, Shan C. Epidemiological investigation on infertility among married women of childbearing age in Shan county, Shandong Province. Chin J Fam Plann. 2002; **10**(3): 150-1(in Chinese).

24. Wang Y, Gao M, Dai L, Zheng Y. Study on the current situation of infertile couples in Zhabei District of Shanghai. Chin J Fam Plann. 2002; **10**(7): 410-3(in Chinese).

25. Hou Q, Jiang H, Huang H. Survey on the infertility in married woman in child - bearing age. Chin Matern Child Health Care. 2007; **22**(36): 5160-4(in Chinese).

26. Cai X, Song R, Long M, Wang S, Ma Y, Li X, et al. A cross-sectional study on the current status of female infertility in three counties of Xinjiang Uygur Autonomous Region. Natl Med J China. 2011; **91**(45): 3182-5(in Chinese).

27. Zhang Y, Jia B, Chang L, Zhao Z, Zhang H, Song X. Epidemiological investigation on infertility reproduction women in Tianjin area. Chin J Fam Plann. 2016; **25**(4): 301-5(in Chinese).

28. Xu M, Yu W, Xing Z, Yao H, Sun J, Yu C. An investigation of reproductive health and related influencing factors in female staff in six industries in seven provinces in China. Chin J Ind Hyg Occup Dis. 2016; **34**(12): 924-7(in Chinese).

29. Zhang Z, Wei X, Du S, Shi Y, Li Q. Investigation and analysis of infertility in Xinmi City. Chinese Rural Health Service Administration. 2002; **22**(4): 55-7(in Chinese).

30. Ou H, Liang Q, Peng C. Analysis on the status and influencing factors of infertility in married couples. Qingdao Medical Journal. 2017; **49**(5): 379-81(in Chinese).

31. Gao J, Gao E. Analysis of infertility rate and its influencing factors among women of childbearing age in China. Chinese Journal of Health Statistics. 2005; **22**(1): 26-8(in Chinese).

32. Yang Y, Xu H, Zha S, LV N, Fu Y, Zhou J, et al. A 5 - year follow - up study of infertility in pre - pregnancy eugenics. Chinese Journal of Family Planning & Gynecotokology. 2020; **12**(1): 30-3(in Chinese).

33. Xing Z, Yu W, Xu M, Yu C. Analysis on infertility status and influencing factors of female workers among reproductive age in China's nine industries. Chin J Prev Med. 2018; **52**(2): 134-40(in Chinese).

34. Li Y. Investigation on the current status of fertility decline in women of childbearing age in Liaoning Province and establishment of a predictive model for ovarian reserve and pregnancy outcome of in vitro fertilization - embryo transfer. Liaoning: Chinese Medical Sciences University; 2021(in Chinese).

35. Wang B. Epidemiological Investigation of infertility in Suzhou Area and TCM syndrome Nanjing: Nanjing University of Chinese Medicine; 2019(in Chinese).

36. Hou L. Epidemiological study on infertility in three provinces of China. Beijing: Peking Union Medical College; 2011(in Chinese).

37. Feng Y. Prevalence and risk factors of infertility in women of childbearing age in Zhejiang Province. Hangzhou: Zhejiang University; 2017(in Chinese).

38. Cong J, Li P, Zheng L, Tan J. Prevalence and Risk Factors of Infertility at a Rural Site of Northern China. PLoS One. 2016; **11**(5): e0155563.

39. Chen J, Zhong C, Liang H, Yang Y, Zhang O, Gao E, et al. The relationship between age at menarche and infertility among Chinese rural women. Eur J Obstet Gynecol Reprod Biol. 2015; **194**: 68-72.

40. Zhou Z, Zheng D, Wu H, Li R, Xu S, Kang Y, et al. Epidemiology of infertility in China: a population-based study. BJOG. 2018; **125**(4): 432-41.

41. Ye Y, Zhang J, Wang Z, Lei W, Hu L, Sun L, et al. Investigation and analysis on fertility among female workers in Beijing. Chinese J Ind Med. 2017; **30**(4): 268-73(in Chinese).

42. Liu S, Wang S, Zhang G, Zhang L, Han H, Wang G, et al. Analysis on the prevalence of primary infertility in rural areas of Huanghua City, Hebei Province. Chin J Fam Plann. 2013; **21**(6): 409-10(in Chinese).

43. An M, Han X, Wang S, Yan J, Xu Y, Mou S, et al. Epidemiologic study of potentia generandi on married women in reproductive age in Rizhao district. J of Pub Health and PreV Med. 2008; **19**(6): 32-4(in Chinese).

44. Zhang Z, Li X, Li B. Ethnic differences in infertility, consanguineous marriage and birth defects in Qinghai --a survey of 1240 couple. Journal of Reproductive Medicine. 2020; **29**(9): 1152-5(in Chinese).

45. Jia Y. Epidemiological investigation of infertility in Qinghai Province. Chin J Public Health. 2004; **20**(10): 1275(in Chinese).

46. Meng Q, Ren A, Zhang L, Liu J, Li Z, Yang Y, et al. Incidence of infertility and risk factors of impaired fecundity among newly married couples in a Chinese population. Reprod Biomed Online. 2015; **30**(1): 92-100.

47. Wei T, Yu W, Mei L, Yao Y, Jiang Z, Zhang H, et al. Survey on reproductive health of married working women in Hubei Province. J of Pub Health and Prev Med. 2018; **29**(5): 44-7(in Chinese).

48. Liu J, Wu S, Xu Y. Investigation of married infertility women of childbearing age in Jiang'an District of Wuhan City and its risk factors. Chin J Fam Plann. 2021; **29**(10): 2036-9(in Chinese).

49. Chen LX, Li R, Ye R. Cumulative live birth rate in vitro fertilization in China: A population-based study. Chinese Journal of Reproductive Health. 2017; **28**(2): 101-5(in Chinese).

50. Hao M. Cumulative live birth rates after in-vitro fertilization and analysis of relative factors. Jinan: Shandong University; 2016(in Chinese).

51. Xu X, Xu C, Chen C, Li J, Liu Y, Zhang W, et al. Influence of abortion in the first complete cycle of assisted reproductive technology on the outcome of subsequent cycles. Journal of Zhengzhou University(Medical Sciences). 2022; **57**(1): 78-82(in Chinese).

52. Yuan L. Treatment Outcomes of Repeated IVF-ET Cycles and Its Influencing Factors. Tianjin: Tianjin Medical University; 2016(in Chinese).

53. Yang J, Zhang X, Ding X, Wang Y, Huang G, Ye H. Cumulative live birth rates between GnRH-agonist long and GnRH-antagonist protocol in one ART cycle when all embryos transferred: real-word data of 18,853 women from China. Reprod Biol Endocrinol. 2021; **19**(1): 124.

54. Huang L, Lu Q, Du J, Lv H, Tao S, Chen S, et al. Cumulative live birth rates of in vitro fertilization/intracytoplasmic sperm injection after multiple complete cycles in China. J Biomed Res. 2020; **34**(5): 361-8.

55. Li Y, Fang Y, Zhang H, Liu L, Gao Y. Cost-effectiveness analysis of frozen-thawed embryos or fresh embryo transfer of IVF in non-PCOS patients with hyper ovarian response. Journal of Reproductive Medicine. 2021; **30**(5): 575-81(in Chinese).

56. Jing M, Lin C, Zhu W, Tu X, Chen Q, Wang X, et al. Cost-effectiveness analysis of GnRH-agonist long-protocol and GnRH-antagonist protocol for in vitro fertilization. Sci Rep. 2020; **10**(1): 8732.

57. Wei X, Wang C, Zhang Y. Comparison of clinical outcome and cost-effectiveness between antagonist and microstimulation protocol among elderly patients with low ovarian reserve function. Chinese Journal of Women and Children Health. 2018; **9**(3): 63-9(in Chinese).

58. Pan W, Tu H, Jin L, Hu C, Li Y, Wang R, et al. Decision analysis about the cost-effectiveness of different in vitro fertilization-embryo transfer protocol under considering governments, hospitals, and patient. Medicine (Baltimore). 2019; **98**(19): e15492.

59. Zhang Y, Bao J, Yao H, Li P, Liu L. Clinical application and economic analysis of gonadotropin-releasing hormone antagonist protocol in patients with decreased ovarian reserve. Chin J Reprod Contracep. 2018; **38**(3): 228-31(in Chinese).

60. Zhu C. Comparative study of clinical outcomes and health economics between Luteal long-acting protocol and GnRH-antagonist protocol in IVF-ET in patients with normal ovarian respone. Kunming: Kunming Medical University; 2021(in Chinese).

61. Zheng J, Zhou L, Sun Y, Xia A, Li M, Liang K. Comparison of the clinical outcomes of three ovulation induction protocols in elderly patients with decline in ovarian reserve. Chin J Reprod Contracep. 2020; **40**(3): 194-200(in Chinese).

62. Li J, Li Y, Nai D. Cost-effectiveness of IVF versus intrauterine insemination. Journal of Reproductive Medicine. 2019; **28**(1): 23-8(in Chinese).

63. Yalin X. IVF-ET outcome and cost-effectiveness in patients with polycystic ovarian syndrome. Zhengzhou: Zhengzhou University; 2016(in Chinese).

64. Dai F, Zheng B, Guo Y, Wang Y, Guo Y, Huo Z, et al. Pregnancy outcome and economic evaluation of different ovulation induction protocols in patients with polycystic ovary syndrome. Journal of Reproductive Medicine. 2020; **29**(9): 1156-61(in Chinese).

65. Dai F, Guo Y, Dong Y, Zheng B, Li H, Guo Y. Comparison of down regulation and non-down regulation in women with advanced age and poor ovarian response. Journal of Reproductive Medicine. 2019; **28**(6): 613-8(in Chinese).

66. Liu Y, Shen C, Yang Y, Zhang Y, Hu J, Wang X. Analysis of the clinical outcomes and economic evaluation of different transferring scheme in IVF/ICSI cycle. Prog Obstet Gynecol. 2017; **26**(4): 283-6(in Chinese).

67. Li Z. New Application of Gamate Intrauterine Transfer in Assisted reproduction and Evaluation of Health Economics. Jinan: Shandong University; 2019(in Chinese).

68. Wu Y, Liu H, Liu J. The Livebirth Rate Per In Vitro Fertilization Cycle Is Higher Than The Cumulative Live Birth Rates of Intrauterine Insemination for Patients of Poseidon Group 3 With Unexplained Infertility. Front Endocrinol (Lausanne). 2021; **12**: 768975.

69. Hao N. Correlations among basal follicle stimulating hormone, antral follicle count and clinical outcomes in patients with advanced age undergoing IVF-ET and health economics analysis. Guangzhou: Southern Medical University 2011(in Chinese).

70. Sun Y, Zhang M, Zhang J, Chang X, Zhu A, Ma X, et al. A study of clinic efficiency on the strategy of super ovulation based on clomiphene citratein IVF. Chinese Journal of Birth Health & Heredity. 2011; **19**(8): 102-5(in Chinese).

71. Ji J, Luo L, Tong X, Luan H, Jin R, Fu Y, et al. Efficacy and economic evaluation of microstimulation in the treatment of ovarian hyporesponsiveness IVF Cycle. Reproduction＆Contraception. 2009; **29**(11): 768-71(in Chinese).

72. Zhang R, Wu B. Clinical Efficacy and Economic Analysis of Three Ovulation Induction Drugs in IVF-ET/ICSI——A Retrospective Analysis Based on Real World. Chin J Mod Appl Pharm. 2021; **38**(17): 2128-33(in Chinese).

73. LV Z. Application of Progestin primed ovarian stimulationregimen in patients with low IVF ovarian reserve. Xining: Qinghai University; 2017(in Chinese).

74. Liu Y, Su R, Wu Y. Cumulative Live Birth Rate and Cost-Effectiveness Analysis of Gonadotropin Releasing Hormone-Antagonist Protocol and Multiple Minimal Ovarian Stimulation in Poor Responders. Front Endocrinol (Lausanne). 2020; **11**: 605939.

75. Lin L, Liu Y, Huang W, Chen G, He L, Mao L, et al. Application of three controlled ovarian hyper‐stimulation protocols in advanced‐age patients with diminished ovarian reserve. Journal of Reproductive Medicine. 2020; **29**(6): 733-8(in Chinese).

76. Pham CT, Karnon JD, Norman RJ, Mol BW. Cost-effectiveness modelling of IVF in couples with unexplained infertility. Reprod Biomed Online. 2018; **37**(5): 555-63.

77. Chambers GM, Sullivan EA, Shanahan M, Ho MT, Priester K, Chapman MG. Is in vitro fertilisation more effective than stimulated intrauterine insemination as a first-line therapy for subfertility? A cohort analysis. Aust N Z J Obstet Gynaecol. 2010; **50**(3): 280-8.

78. Chambers GM, Ho MT, Sullivan EA. Assisted reproductive technology treatment costs of a live birth: an age-stratified cost-outcome study of treatment in Australia. Med J Aust. 2006; **184**(4): 155-8.

79. Fragoulakis V, Maniadakis N. Estimating the long-term effects of in vitro fertilization in Greece: an analysis based on a lifetime-investment model. Clinicoecon Outcomes Res. 2013; **5**: 247-55.

80. Maldonado LG, Franco JG, Jr., Setti AS, Iaconelli A, Jr., Borges E, Jr. Cost-effectiveness comparison between pituitary down-regulation with a gonadotropin-releasing hormone agonist short regimen on alternate days and an antagonist protocol for assisted fertilization treatments. Fertil Steril. 2013; **99**(6): 1615-22.

81. De Sutter P, Gerris J, Dhont M. A health-economic decision-analytic model comparing double with single embryo transfer in IVF/ICSI. Hum Reprod. 2002; **17**(11): 2891-6.

82. Strandell A, Lindhard A, Eckerlund I. Cost--effectiveness analysis of salpingectomy prior to IVF, based on a randomized controlled trial. Hum Reprod. 2005; **20**(12): 3284-92.

83. Xue W, Lloyd A, Falla E, Roeder C, Papsch R, Bühler K. A cost-effectiveness evaluation of the originator follitropin alpha compared to the biosimilars for assisted reproduction in Germany. Int J Womens Health. 2019; **11**: 319-31.

84. Gizzo S, Ferrando M, Lispi M, Ripellino C, Cataldo N, Bühler K. A cost-effectiveness modeling evaluation comparing a biosimilar follitropin alfa preparation with its reference product for live birth outcome in Germany, Italy and Spain. J Med Econ. 2018; **21**(11): 1096-101.

85. Ferrier C, Boujenah J, Poncelet C, Chabbert-Buffet N, Mathieu D'Argent E, Carbillon L, et al. Use of the EFI score in endometriosis-associated infertility: A cost-effectiveness study. Eur J Obstet Gynecol Reprod Biol. 2020; **253**: 296-303.

86. Barriere P, Porcu-Buisson G, Hamamah S. Cost-Effectiveness Analysis of the Gonadotropin Treatments HP-hMG and rFSH for Assisted Reproductive Technology in France: A Markov Model Analysis. Appl Health Econ Health Policy. 2018; **16**(1): 65-77.

87. Veleva Z, Karinen P, Tomás C, Tapanainen JS, Martikainen H. Elective single embryo transfer with cryopreservation improves the outcome and diminishes the costs of IVF/ICSI. Hum Reprod. 2009; **24**(7): 1632-9.

88. Chambers GM, Sullivan EA, Ishihara O, Chapman MG, Adamson GD. The economic impact of assisted reproductive technology: a review of selected developed countries. Fertil Steril. 2009; **91**(6): 2281-94.

89. Lukassen HG, Braat DD, Wetzels AM, Zielhuis GA, Adang EM, Scheenjes E, et al. Two cycles with single embryo transfer versus one cycle with double embryo transfer: a randomized controlled trial. Hum Reprod. 2005; **20**(3): 702-8.

90. Groen H, Tonch N, Simons AH, van der Veen F, Hoek A, Land JA. Modified natural cycle versus controlled ovarian hyperstimulation IVF: a cost-effectiveness evaluation of three simulated treatment scenarios. Hum Reprod. 2013; **28**(12): 3236-46.

91. Fragoulakis V, Pescott CP, Smeenk JM, van Santbrink EJ, Oosterhuis GJ, Broekmans FJ, et al. Economic Evaluation of Three Frequently Used Gonadotrophins in Assisted Reproduction Techniques in the Management of Infertility in the Netherlands. Appl Health Econ Health Policy. 2016; **14**(6): 719-27.

92. van Tilborg TC, Oudshoorn SC, Eijkemans MJC, Mochtar MH, van Golde RJT, Hoek A, et al. Individualized FSH dosing based on ovarian reserve testing in women starting IVF/ICSI: a multicentre trial and cost-effectiveness analysis. Hum Reprod. 2017; **32**(12): 2485-95.

93. Polinder S, Heijnen EM, Macklon NS, Habbema JD, Fauser BJ, Eijkemans MJ. Cost-effectiveness of a mild compared with a standard strategy for IVF: a randomized comparison using cumulative term live birth as the primary endpoint. Hum Reprod. 2008; **23**(2): 316-23.

94. Vélez MP, Connolly MP, Kadoch IJ, Phillips S, Bissonnette F. Universal coverage of IVF pays off. Hum Reprod. 2014; **29**(6): 1313-9.

95. Vitek WS, Galárraga O, Klatsky PC, Robins JC, Carson SA, Blazar AS. Management of the first in vitro fertilization cycle for unexplained infertility: a cost-effectiveness analysis of split in vitro fertilization-intracytoplasmic sperm injection. Fertil Steril. 2013; **100**(5): 1381-8.

96. Murugappan G, Ohno MS, Lathi RB. Cost-effectiveness analysis of preimplantation genetic screening and in vitro fertilization versus expectant management in patients with unexplained recurrent pregnancy loss. Fertil Steril. 2015; **103**(5): 1215-20.

97. Kansal-Kalra S, Milad MP, Grobman WA. In vitro fertilization (IVF) versus gonadotropins followed by IVF as treatment for primary infertility: a cost-based decision analysis. Fertil Steril. 2005; **84**(3): 600-4.

98. Lee M, Lofgren KT, Thomas A, Lanes A, Goldman R, Ginsburg ES, et al. The cost-effectiveness of preimplantation genetic testing for aneuploidy in the United States: an analysis of cost and birth outcomes from 158,665 in vitro fertilization cycles. Am J Obstet Gynecol. 2021; **225**(1): 55 e1- e17.

99. Maeda E, Ishihara O, Saito H, Kuwahara A, Toyokawa S, Kobayashi Y. Age-specific cost and public funding of a live birth following assisted reproductive treatment in Japan. J Obstet Gynaecol Res. 2014; **40**(5): 1338-44.

100. Almaslami F, Aljunid SM. Cost-effectiveness of assisted reproductive technologies in Saudi Arabia: Comparing in vitro fertilization with intrauterine insemination. SAGE Open Med. 2020; **8**: 2050312120931988.

101. Rafique M, Al-Badr A, Saleh A, Al-Jaroudi DH. Economic perspective of evaluating fertility treatment in obese and overweight infertile women. Saudi Med J. 2021; **42**(6): 666-72.

102. Yildiz MS, Khan MM. Opportunities for reproductive tourism: cost and quality advantages of Turkey in the provision of in-vitro Fertilization (IVF) services. BMC Health Serv Res. 2016; **16**(a): 378.

103. Hernandez Torres E, Navarro-Espigares JL, Clavero A, López-Regalado M, Camacho-Ballesta JA, Onieva-García M, et al. Economic evaluation of elective single-embryo transfer with subsequent single frozen embryo transfer in an in vitro fertilization/intracytoplasmic sperm injection program. Fertil Steril. 2015; **103**(3): 699-706.

104. Papaleo E, Pagliardini L, Vanni VS, Delprato D, Rubino P, Candiani M, et al. A direct healthcare cost analysis of the cryopreserved versus fresh transfer policy at the blastocyst stage. Reprod Biomed Online. 2017; **34**(1): 19-26.

105. Levi-Setti PE, Busnelli A, De Luca R, Scaravelli G. Do Strategies Favoring Frozen-thawed Embryo Transfer Have an Impact on Differences in IVF Success Rate, Multiple Pregnancy Rate, and Cost per Live Birth Between Fertility Clinics? Reprod Sci. 2022; **29**(4): 1379-86.

106. Wechowski J, Connolly M, Schneider D, McEwan P, Kennedy R. Cost-saving treatment strategies in in vitro fertilization: a combined economic evaluation of two large randomized clinical trials comparing highly purified human menopausal gonadotropin and recombinant follicle-stimulating hormone alpha. Fertil Steril. 2009; **91**(4): 1067-76.

107. Maheshwari A, Scotland G, Bell J, McTavish A, Hamilton M, Bhattacharya S. The direct health services costs of providing assisted reproduction services in overweight or obese women: a retrospective cross-sectional analysis. Hum Reprod. 2009; **24**(3): 633-9.

108. Braam SC, Ho VNA, Pham TD, Mol BW, van Wely M, Vuong LN. In-vitro maturation versus IVF: a cost-effectiveness analysis. Reprod Biomed Online. 2021; **42**(1): 143-9.

109. Le KD, Vuong LN, Ho TM, Dang VQ, Pham TD, Pham CT, et al. A cost-effectiveness analysis of freeze-only or fresh embryo transfer in IVF of non-PCOS women. Hum Reprod. 2018; **33**(10): 1907-14.

110. Khoa LD, Lan VTN, Loc NMT, Vinh DQ, Tran QN, Tuong HM. Corifollitropin alfa versus follitropin beta: an economic analysis alongside a randomized controlled trial in women undergoing IVF/ICSI. Reprod Biomed Soc Online. 2020; **10**: 28-36.

111. Widge A. Seeking conception: experiences of urban Indian women with in vitro fertilisation. Patient Educ Couns. 2005; **59**(3): 226-33.

112. Sini I, Polim AA, Handayani N, Pratiwi A, Thuffi R, Yusup N, et al. Embryo Banking with Mild Ovarian Stimulation for IVF: An Alternative Strategy for Poor Prognosis Patients. J Reprod Infertil. 2020; **21**(3): 176-82.

113. Saraeva NV, Spiridonova NV, Tugushev MT, Shurygina OV, Arabadzhyan SI, Victorovna IO. Experience of using time lapse microscopy in the IVF program in patients with good ovarian reserve. Gynecol Endocrinol. 2019; **35**(sup1): 15-7.
