# Supplementary material for: Feasibility analysis of China's medical insurance coverage of assisted reproductive technology
Source: Sci Rep. 2024 Apr 5;14:7998. doi: 10.1038/s41598-024-58640-4 (PMC10997767; doi:10.1038/s41598-024-58640-4)
Supplement: Supplementary file 4 — Supplementary Information 4. [file 41598_2024_58640_MOESM4_ESM.docx]

**Supplementary material 4**

**Partial cost list for IVF/ICIS treatment**

| **Cost List** | **Price** | **Literature** |
| --- | --- | --- |
| **Drugs** |  |  |
| Triptorelin (0.1 mg) | 105 ~ 163 | [1-2,4-5] |
| Triptorelin (3.75 mg) | 1280 ~ 1864 | [3-5] |
| Cetrorelix Acetate Powder for Injection (0.25mg) | 345 ~ 422 | [3,5] |
| Recombinant human follicle stimulating hormone (Gonal-F 450IU) | 1496 ~ 1739 | [1-4] |
| HMG (75IU) | 20 ~ 33 | [1-5] |
| rFSH (75IU) | 240 ~ 300 | [1,4-5] |
| uFSH (75IU) | 123 ~ 150 | [3-5] |
| Cetrorelix (0.25 mg) | 343 | [1-2] |
| Ovidrel (6500IU) | 192 | [1-2] |
| Dydrogesterone tablets (tablet) | 5 | [1-2] |
| Progesterone vaginal sustained release gel %8 (dose) | 69 | [1] |
| Progesterone capsules (capsule) | 3 | [2] |
| HCG (5000IU) | 27 | [3] |
| Recombinant Human Lutropin alfa (75IU) | 242 | [3] |
| Recombinant follicle stimulating hormone β (50IU) | 191 | [3] |
| **Operation** |  |  |
| Oocyte retrieval | 1410 ~ 2000 | [2-5] |
| Semen optimization | 380 ~ 675 | [1,4] |
| Embryo culture | 3000 ~ 4000 | [1,2-5] |
| Blastocyst culture | 1500 | [1] |
| Embryo transplantation | 100 ~ 1600 | [2-5] |
| Embryo cryopreservation | 3000 ~ 4000 | [1-2] |
| Frozen embryo preservation (1 tube * year) | 800 ~ 1500 | [3,5] |
| Embryos thawing | 1200 | [1] |
| Frozen-thawed embryo transfer | 2180 | [3] |
| Anesthetic Fee | 624 ~ 1000 | [2,4] |
| IVF | 1300 ~ 2000 | [2,4,5] |
| ICIS | 3000 | [4] |
| **Examination** |  |  |
| B-ultrasound | 78 ~ 80 | [1,4] |
| Color Doppler ultrasound | 180 | [4] |
| Serum FSH examination | 30 ~ 60 | [1-4] |
| Serum HCG examination | 30 ~ 60 | [1-2,4] |
| Serum LH examination | 30 ~ 60 | [1-4] |
| Serum E2 examination | 30 ~ 60 | [1-4] |
| Serum P examination | 30 ~ 60 | [1-4] |
| Sperm morphology examination | 143 | [5] |
| Sperm acrosin and fructose examination | 147 | [5] |

Note: HMG-human menopausal gonadotropin; rFSH-Recombinant follicle stimulating hormone; uFSH-Urinary follicle stimulating hormone; HCG-Chorionic Gonadotrophin; HCM-Human menopausal gonadotrophin; LH-Luteinizing hormone; E2-Estradiol; P-pregnendione.

**References**

[1] Li Y, Fang Y, Zhang H, Liu L, Gao Y. Cost-effectiveness analysis of frozen-thawed embryos or fresh embryo transfer of IVF in non-PCOS patients with hyper ovarian response. Journal of Reproductive Medicine. 2021; 30(5): 575-81(in Chinese)

[2] Jing M, Lin C, Zhu W, Tu X, Chen Q, Wang X, et al. Cost-effectiveness analysis of GnRH-agonist long-protocol and GnRH-antagonist protocol for in vitro fertilization. Sci Rep. 2020; 10(1): 8732.

[3] Zhu C. Comparative study of clinical outcomes and health economics between Luteal long-acting protocol and GnRH-antagonist protocol in IVF-ET in patients with normal ovarian respone. Kunming: Kunming Medical University; 2021(in Chinese).

[4] Yalin X. IVF-ET outcome and cost-effectiveness in patients with polycystic ovarian syndrome. Zhengzhou: Zhengzhou University; 2016(in Chinese).

[5] Hao N. Correlations among basal follicle stimulating hormone, antral follicle count and clinical outcomes in patients with advanced age undergoing IVF-ET and health economics analysis. Guangzhou: Southern Medical University 2011(in Chinese)
